# Stem Photosynthesis—A Key Element of Grass Pea (*Lathyrus sativus* L.) Acclimatisation to Salinity

**DOI:** 10.3390/ijms22020685

**Published:** 2021-01-12

**Authors:** Krzysztof M. Tokarz, Wojciech Wesołowski, Barbara Tokarz, Wojciech Makowski, Anna Wysocka, Roman J. Jędrzejczyk, Karolina Chrabaszcz, Kamilla Malek, Anna Kostecka-Gugała

**Affiliations:** 1 Department of Botany, Physiology and Plant Protection, Faculty of Biotechnology and Horticulture, University of Agriculture in Krakow, 29 Listopada 54, 31-425 Krakow, Poland; barbara.tokarz@urk.edu.pl (B.T.); wojtek.makowski.1305@gmail.com (W.M.); wysocka.anna.magdalena@gmail.com (A.W.); 2 Department of Plant Biology and Biotechnology, Faculty of Biotechnology and Horticulture, University of Agriculture in Krakow, 29 Listopada 54, 31-425 Krakow, Poland; w.wesolowski@ogr.ur.krakow.pl (W.W.); anna.kostecka-gugala@urk.edu.pl (A.K.-G.); 3 Centre Algatech, Institute of Microbiology, Czech Academy of Sciences, 379 81 Třeboň, Czech Republic; 4 Plant-Microorganism Interactions Group, Malopolska Centre of Biotechnology, Jagiellonian University, Gronostajowa 7A, 30-387 Krakow, Poland; roman.jedrzejczyk@uj.edu.pl; 5 Raman Imaging Group, Faculty of Chemistry, Jagiellonian University, Gronostajowa 2, 30-387 Krakow, Poland; karolina.chrabaszcz@doctoral.uj.edu.pl (K.C.); kamilla.malek@uj.edu.pl (K.M.)

**Keywords:** *Lathyrus sativus*, cyclic electron transport, linear electron transport, photosynthetic apparatus, photosystem I, photosystem II, ROS, salt stress

## Abstract

Grass pea (*Lathyrus sativus*) is a leguminous plant of outstanding tolerance to abiotic stress. The aim of the presented study was to describe the mechanism of grass pea (*Lathyrus sativus* L.) photosynthetic apparatus acclimatisation strategies to salinity stress. The seedlings were cultivated in a hydroponic system in media containing various concentrations of NaCl (0, 50, and 100 mM), imitating none, moderate, and severe salinity, respectively, for three weeks. In order to characterise the function and structure of the photosynthetic apparatus, Chl *a* fluorescence, gas exchange measurements, proteome analysis, and Fourier-transform infrared spectroscopy (FT-IR) analysis were done inter alia. Significant differences in the response of the leaf and stem photosynthetic apparatus to severe salt stress were observed. Leaves became the place of harmful ion (Na^+^) accumulation, and the efficiency of their carboxylation decreased sharply. In turn, in stems, the reconstruction of the photosynthetic apparatus (antenna and photosystem complexes) activated alternative electron transport pathways, leading to effective ATP synthesis, which is required for the efficient translocation of Na^+^ to leaves. These changes enabled efficient stem carboxylation and made them the main source of assimilates. The observed changes indicate the high plasticity of grass pea photosynthetic apparatus, providing an effective mechanism of tolerance to salinity stress.

## 1. Introduction

Salinity is one of the main causes of soil degradation in the world and the most common abiotic stress affecting crop plants [1,2]. It is estimated that more than 6% of land area and 20% of irrigated areas in the world are affected by salinisation, and the problem still intensifies [3,4]. The production of food for the ever-growing human population demands an extension of the acreage of arable fields. Frequently, these areas require intensive irrigation, due to insufficient precipitation and/or saline soils, which, combined with the increasing deficit of fresh water, will impose use of seawater and, in consequence, increase the salinity problem [5,6].

Plants that are exposed to salinity have to cope with osmotic and ionic stress. The former is induced by a reduction of the soil water potential, and the latter results from the accumulation of Na^+^ and Cl^−^ in toxic amounts [4,7,8]. The reduced soil water potential makes water unavailable for plants and leads to decreased cell water content and turgor loss [9,10]. Although the reduced cell volume is temporary, it causes growth retardation [9,11]. This is not a specific reaction to salinity; the same changes occur in plants that are subjected to other abiotic stressors, such as drought or cold, which leads to dehydration [9,11,12,13]. In turn, specific reactions to salinity are those connected by the presence of Na^+^ and Cl^−^ ions. Although the plant can accumulate a certain number of harmful ions in the vacuole, the excess concentration of these ions effectively limits the vacuole’s compartmentalisation ability. The result is an accumulation of harmful ions in the cytoplasm and, consequently, an adverse effect on cellular metabolism [7,14]. The cytotoxic effects of Na^+^ results from: (1) a high charge-to-weight ratio that disrupts the water structure and reduces hydrophobic interactions in proteins, thus contributing to their destabilisation; and, (2) binding to enzyme inhibition sites by means of non-competitive inhibition or replacing K^+^ ions at the active site of the enzyme by means of competitive inhibition [14]. In both cases, competition between Na^+^ and K^+^ plays a key role; therefore, toxicity may result not only from the concentration of Na^+^ ions, but also from the ratio of Na^+^ and K^+^ ions in the cytosol [15]. The ions that accumulated in the roots can freely move along with the transpiration current and reach the leaves, where they negatively affect photosynthesis—the most important process for plant life [4,7]. Photosynthesis efficiency depends on the sequence of metabolic processes during light and dark photosynthesis reactions, activity of enzymes that are involved in carbon assimilation, the structure of the photosynthetic apparatus, and the transport of photosynthetic intermediates between cellular compartments [16]. Harmful ions can accumulate in chloroplasts, causing a direct toxic effect on the photosynthesis process by the degradation of photosynthetic pigments [17]. A decrease of the chlorophyll concentration in leaves, as well as disorganisation of the thylakoid membrane structure and reduction of thylakoid and grana number, were observed in various plant species that are sensitive to salt stress [16,18,19]. Moreover, salt stress destabilises protein complexes of the photosynthetic electron transport chain, such as photosystem II (PSII) with the oxygen evolving complex (OEC), photosystem I (PSI), the cytochrome *b6f* complex, and ATP synthase [20,21,22]. Indirectly, salinity significantly affects the availability of carbon dioxide (by limiting CO_2_ diffusion to the chloroplast due to closed stomata) and water [23]. The reduced intercellular CO_2_ (Ci) under salinity limits the activity of Calvin–Benson cycle enzymes, especially ribulose bisphosphate carboxylase/oxygenase (RuBisCo) [16,24,25]. Salinity very often also leads to the photoinhibition process, because of reduced photosynthetic efficiency without a reduced intensity of photosynthetically active radiation (PAR) [26]. The excess of untapped energy damages the photosynthetic apparatus, which, in consequence, generates reactive oxygen species (ROS) [26,27]. Excessive concentrations of ROS in the cell, with insufficient antioxidant activity, can lead to lipid peroxidation, resulting in changes of membrane structure and its physical properties, such as permeability to various compounds [28]. Other molecules that are sensitive to ROS are proteins, including enzymes and photosynthetic proteins, and DNA [24,29]. ROS also contribute to decrease the concentration of K^+^ ions in the cytosol [29]. Plants developed protective mechanisms in the form of enzymatic and non-enzymatic antioxidants in order to avoid excessive accumulation of ROS during stress [29,30]. Non-enzymatic antioxidants include ascorbic acid, glutathione, carotenoids, flavonoids, and other phenolic compounds, among others [31]. The most common antioxidant enzyme systems are superoxide dismutase (SOD), catalase (CAT), ascorbic peroxidase (APX), and other peroxidases [31]. Under unfavourable conditions for plant growth and development, both the biosynthesis of non-enzymatic antioxidant molecules and enzyme activity change [29,30].

The tolerance and response mechanisms to salinity vary depending on the plant species [32]. Grass pea (*Lathyrus sativus* L.) is a plant grown successfully in regions that are struggling with the problem of drought where other legumes do not yield sufficiently high [33]. In addition, grass pea exhibits resistance to other abiotic stressors, such as moderate salinity and periodic flooding [34,35]. This unique, among other legumes, resistance to abiotic stress, combined with the favourable composition of seeds, makes the grass pea both a future crop of sustainable agriculture and an interesting object of basic research.

The aim of this study was to describe the mechanism of grass pea (*Lathyrus sativus* L.) photosynthetic apparatus response to salinity stress. We hypothesised that: (1) the acclimatisation of plants to salt stress would be associated with the reconstruction of the photosynthetic apparatus, including the antenna system with photosystems and the electron transporter system; and, (2) this reconstruction would enable the effective transport of electrons, thus reducing the risk of photooxidation and providing the possibility of effective CO_2_ carboxylation.

Our results revealed significant differences in the leaf and stem photosynthetic apparatus response to salt stress. Under severe salt stress, the efficiency of leaf carboxylation decreased sharply. Moreover, analyses of ion distribution disclosed that leaves were turned into the place of harmful ion (Na^+^) accumulation. In contrast, the acclimatisation mechanisms in the stems led to the reconstruction of the photosynthetic apparatus (antenna and photosystem complexes), enabling efficient ATP synthesis being utilised for Na^+^ translocation from stems to leaves, as well as efficient carboxylation. Consequently, the stems became the main source of assimilates.

## 2. Results

### 2.1. Growth of Grass Pea Seedlings under Salinity

**Figure 1:**
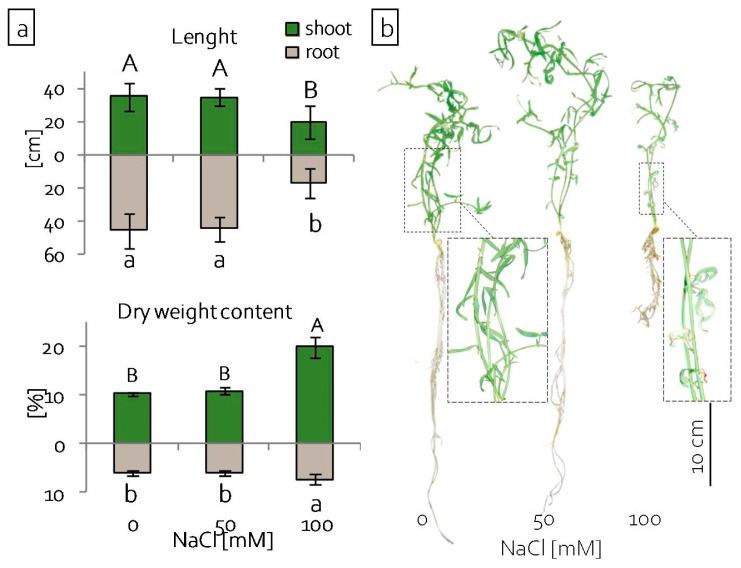
Reaction of grass pea seedlings to salinity stress: (**a**) length (*n* = 30) and percentage of dry weight content of shoots and roots (*n* = 5); and, (**b**) morphology of shoots and roots; different letters—statistically significant differences within each organ (shoot-uppercase, root-lowercase) at *p* ≤ 0.05.

Six days after sowing the seeds, emerging seedlings were transferred to a hydroponic system with media of the corresponding NaCl concentration. In order to evaluate the effect of salinity stress on the growth and development of plants, the length of shoots and percentage of dry weight content were assessed after 25 days of cultivation. The growth of seedlings, both shoots and roots, was only significantly reduced in media with the highest tested NaCl concentration (100 mM) (Figure 1a,b). The percentage of dry weight content increased significantly in both the shoots and roots of these seedlings (Figure 1a). The effect of stress was also reflected in leaf morphology. After 20 days of culture, the leaves of seedlings grown in 100 mM NaCl became discoloured and withered. At the same time, the stems of these plants remained green and alive (Figure 1b).

### 2.2. Reaction of Grass Pea Leaves and Stems to Salinity

**Figure 2:**
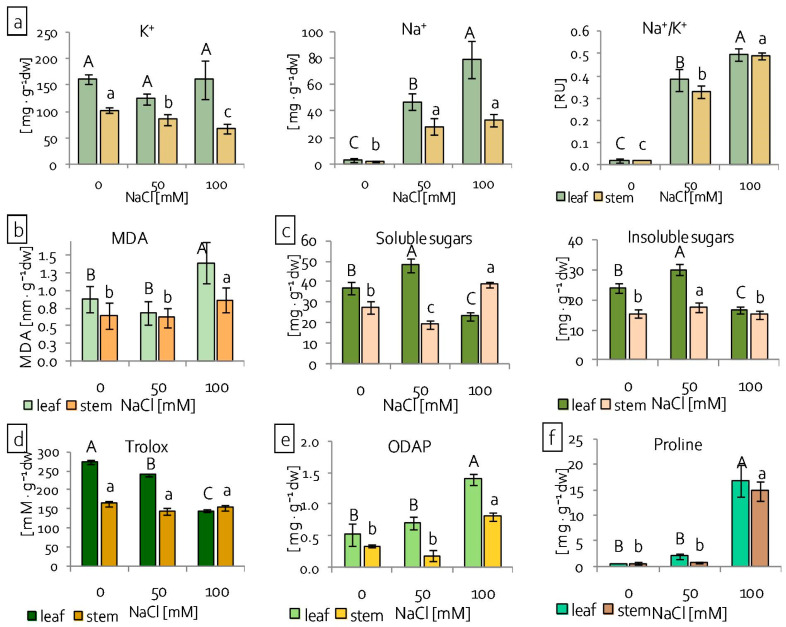
Reaction of grass pea leaves and stems to salinity stress: (**a**) distribution of K^+^ and Na^+^ ions; (**b**) malondialdehyde (MDA) content; (**c**) content of soluble and insoluble sugars; (**d**) total antioxidant capacity (as Trolox equivalents); (**e**) content of β-N-oxalyl-L-α,β-diamino propionic acid (ODAP); and, (**f**) content of proline; different letters—statistically significant differences within each organ (leaf-uppercase, stem-lowercase) at *p* ≤ 0.05; (*n* = 5).

The results that are presented above disclosed a slight effect of salinity on grass pea seedling roots and significant translocation of Na^+^ ions to the shoots. Further analyses were conducted on the leaves and stems separately in order to describe how grass pea shoots respond to an excess of harmful ions. Analyses of ion content revealed different distribution patterns of potassium and sodium ions in leaves and stems, depending on the NaCl concentration. In leaves, the content of K^+^ did not change, regardless of NaCl concentration, but, in stems, it decreased significantly with the increasing NaCl concentration (Figure 2a). In turn, Na ion content increased significantly with increasing NaCl concentrations in leaves of grass pea seedlings, whereas, in stems, the Na^+^ content increased under both NaCl treatments to the same level (Figure 2a). The rate of Na^+^/K^+^ ions increased significantly in both of the tested organs (Figure 2a). The integrity of cell membranes was evaluated by assessing the level of malondialdehyde (MDA)—a product of lipid peroxidation [36]. The lipid peroxidation level increased both in leaves and stems under higher NaCl concentrations, as seen in increased MDA accumulation (Figure 2b). However, the degree of MDA accumulation was greater in leaves than in stems and in relation to the control, which increased 60% and 36%, respectively (Figure 2b). The antioxidant capacity of the seedlings was determined while using the ferric reducing antioxidant power (FRAP) assay, which enables the assessment of low molecular antioxidant activity, including phenols, ascorbic acid, and simple sugars [37,38]. Antioxidant capacity analyses, done separately in leaves and stems, revealed differences between the reactions of these organs to salinity (Figure 2d). While, in the leaves, the antioxidant capacity decreased with increasing NaCl concentration, it did not change in the stems. Analyses of soluble and insoluble sugars, β-N-oxalyl-L-α,β-diamino propionic acid (ODAP) and proline accumulation, were conducted in order to determine osmoregulatory and osmoprotective responses of seedling leaves and stems. In the leaves, both soluble and insoluble sugar accumulation increased under 50 mM NaCl treatment and decreased under 100 mM NaCl treatment (Figure 2c). In the stems, under lower NaCl concentration, soluble sugar accumulation was reduced; however, the insoluble sugar content was increased (Figure 2c). The higher NaCl concentration increased soluble sugar content and did not change the insoluble sugar content in the stems (Figure 2c). In both of the examined organs, ODAP accumulation increased significantly, more than two-fold, under severe (100 mM NaCl) salinity stress in comparison to the control (Figure 2e). Similarly, proline accumulation only increased significantly in organs of seedlings growing under 100 mM NaCl stress (Figure 2f). However, the content of proline was almost thirty times higher than in organs of the control plants (Figure 2f).

**Figure 3:**
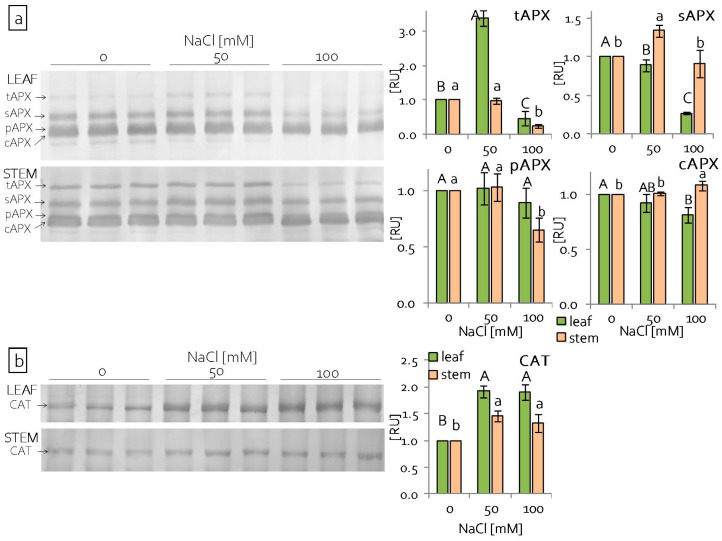
Content of antioxidant enzymes: (**a**) APX isoforms and (**b**) CAT in leaves and stems of grass pea shoots under salinity; tAPX—thylakoid ascorbate peroxidase, sAPX—stromal ascorbate peroxidase, pAPX—peroxisomal ascorbate peroxidase, cAPX—cytoplasmic ascorbate peroxidise, CAT—catalase; content of proteins expressed as relative units [RU]; different letters—statistically significant differences within each organ (leaf-uppercase, stem-lowercase) at *p* ≤ 0.05 (*n* = 3).

Additionally, analyses of antioxidant enzyme content while using SDS-PAGE and immunoblotting were performed. Four isoforms of ascorbate peroxidise (APX) were detected: thylakoid (t), stromal (s), peroxisomal (p), and cytoplasmic (c) (Figure 3a). The content of tAPX increased significantly in leaves under the lower NaCl concentration used and decreased, both in the leaves and stems, under the higher NaCl concentration (Figure 3a). Stromal APX content decreased in grass pea leaves and it did not change in stems under severe salinity, in comparison to the control plants. In turn, pAPX content decreased in the stems of grass pea seedlings that were treated with 100 mM NaCl. The content of cAPX decreased under severe salinity in the leaves, but increased in the stems (Figure 3a). In contrast to the ascorbate peroxidise content, which generally decreased under salinity stress, the content of catalase increased in both the leaves and stems, regardless of NaCl concentration (Figure 3b).

### 2.3. Leaf and Stem Photosynthetic Apparatus Performance under Salinity Stress

**Figure 4:**
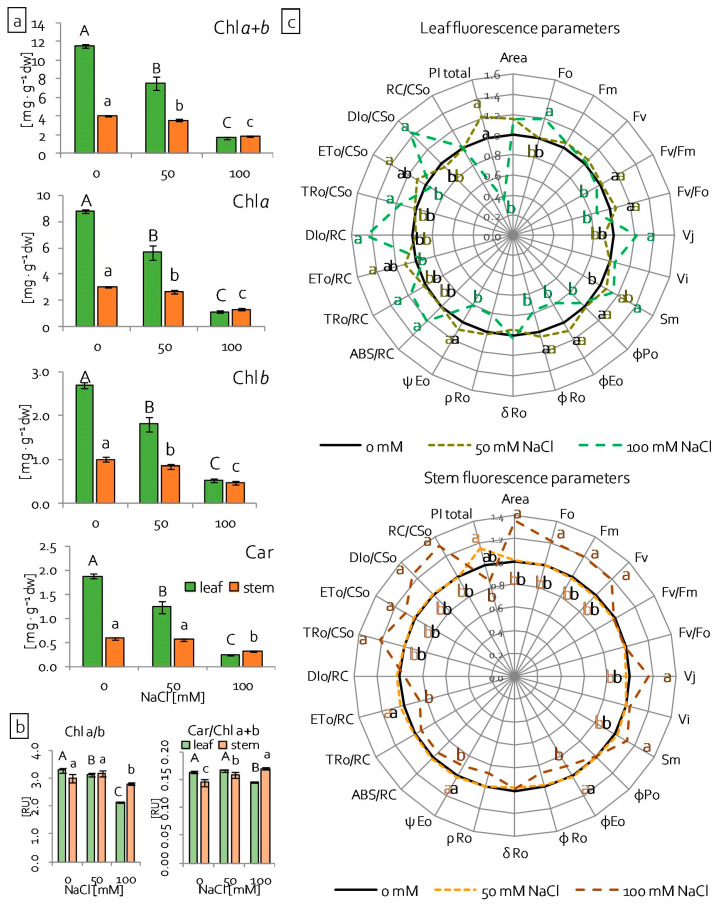
Reaction of the grass pea leaf and stem photosynthetic apparatus to salinity: (**a**) photosynthetic pigment content (*n* = 5); (**b**) pigment ratios (*n* = 5); (**c**) structural and functional parameters of PSII (*n* = 10); Chl *a + b*—total chlorophylls, Chl *a*—chlorophyll *a*, Chl *b*—chlorophyll *b*, Car—carotenoids; all of the values in c were expressed relative to the control (set as 1); abbreviations—see chapter 4.3.2.; RU—relative units; different letters—statistically significant differences within each organ (leaf-uppercase, stem-lowercase) at *p* ≤ 0.05.

Basic parameters of their efficiency were evaluated in order to determine how salinity affects leaf and stem photosynthesis and the photosynthetic apparatus. As an integral element of the photosynthetic apparatus, changes in photosynthetic pigment (chlorophylls Chl and carotenoids Car) content is one of the indicators of its performance [16]. In grass pea leaves and stems, the content of Chl pigments (Chl *a*, Chl *b*, Chl *a + b*) decreased gradually with increasing NaCl concentration (Figure 4a). Nevertheless, in the stems, the Chl content decreased from 12 to 16% under 50 mM NaCl and from 43 to 50% under 100 mM NaCl, in comparison to the control. The reduction was greater in the leaves. It was from 33 to 36% under 50 mM NaCl and from 70 to 80% under 100 mM NaCl (Figure 4a). The car content was significantly reduced in leaves, almost 35% under 50 mM NaCl and 80% under 100 mM NaCl in comparison to the control (Figure 4a). In stems, the Car content decreased by about half only under the higher NaCl concentration (Figure 4a). Additionally, significant alterations were noted in the pigments’ ratios under salinity stress (Figure 4b). The Chl *a*/Chl *b* ratio decreased significantly in leaves with increasing NaCl concentration and in stems, under 100 mM NaCl (Figure 4b), whereas the Car/Chl *a + b* ratio decreased in leaves under the higher NaCl concentration (Figure 4b). However, it increased gradually with increasing NaCl concentration in stems (Figure 4b). Another good indicator of the photosynthetic apparatus response to stress is the measurement of Chl *a* fluorescence, which allowed for the determination of PSII photochemistry efficiency [39,40,41]. The structural and functional photosynthetic parameters of PSII (see Material and methods) were calculated based on the chlorophyll fluorescence induction curve (Supplementary Materials: Figure S1) [42,43,44], both in leaves and stems of grass pea seedlings. Generally, the estimated parameters did not change significantly in comparison to the control, both in leaves and stems, under milder salinity stress (50 mM NaCl) (Figure 4c). Conversely, significant differences in the parameters were noted under 100 mM NaCl (Figure 4c). Minimal fluorescence (Fo) significantly increased in both leaves and stems, but maximal (Fm) and variable (Fv) fluorescence only increased in stems and did not change in leaves (Figure 4c). The maximum quantum yield of PSII (Fv/Fm) and efficiency of the OEC on the donor side of PSII (Fv/Fo) decreased in leaves and it did not change in stems (Figure 4c, Supplementary Materials: Table S1). OJIP parameters, relative variable fluorescence at 2 ms—J-step (V_J_) and 30 ms—I-step (V_I_), and normalised total complementary area above the OJIP transient (Sm) changed the same in leaves and stems under 100 mM NaCl. V_J_ and Sm increased and V_I_ did not change (Figure 4c). Most of the quantum yields and flux ratio parameters (φPo, φEo, ψEo, φRo, ρRo) were reduced in both leaves and stems. However, in stems, a significant decrease was noted in the efficiency of electron transfer from Q_A‾_ to the electron transport chain beyond (φEo), as well as the probability that trapped excitons will move an electron into the electron transport chain beyond Q_A_ (ψEo) (Figure 4c). Specific fluxes per reaction centre (RC) changed differentially in the leaves and stems under 100 mM NaCl. In leaves, the absorption flux per RC (ABS/RC), trapped energy flux per RC (TRo/RC), and dissipated energy flux per RC (DIo/RC) increased in leaves, and it did not change in stems (Figure 4c). However, electron transport flux per RC (ETo/RC) decreased in stems and it did not change in leaves in comparison to the control (Figure 4c). Phenomenological fluxes, trapped energy, and dissipated energy fluxes per excited cross section (CS) increased in both leaves and stems under higher NaCl concentration (Figure 4c). Electron transport flux per CS (ETo/CS) increased only in stems (Figure 4c). The amount of active PSII RC (RC/CSo) and plastoquinone pool (Area) increased significantly only in stems (Figure 4c). The total performance index (PI_Total_) only decreased significantly in the leaves of seedlings growing under 100 mM NaCl (Figure 4c).

**Figure 5:**
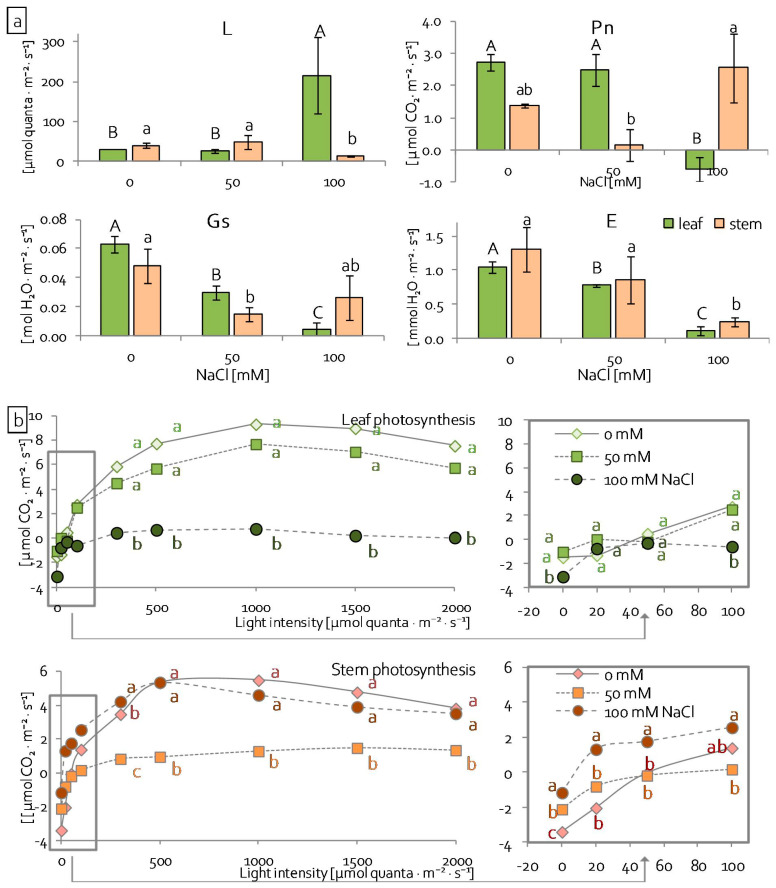
Efficiency of grass pea leaf and stem photosynthesis under salinity: (**a**) light compensation point (L), net photosynthesis (Pn), stomatal conductance (Gs), and transpiration (E) of leaves and stems at 100 μmol quanta·m^−2^·s^−1^; (**b**) leaf and stem photosynthesis efficiency; different letters—statistically significant differences within each organ (leaf-uppercase, stem-lowercase) at *p* ≤ 0.05; (*n* = 3).

In order to verify how salinity affects photosynthesis efficiency, gas exchange measurements in infrared were performed. Measurements were used to determine the light compensation point (L), actual photosynthesis efficiency (Pn) (at 100 μmol quanta·m^−2^·s^−1^), stomatal conductance (Gs), and transpiration (E). The intensity of light when the rate of photosynthesis matched the rate of respiration (net CO_2_ assimilation = 0), i.e., light compensation point was significantly higher in leaves of seedlings treated with 100 mM NaCl (215.5 μmol quanta·m^−2^·s^−1^) than in the control (29.2 μmol quanta·m^−2^·s^−1^) (Figure 5a). In stems under the same stress condition, the light compensation point was reached at 14.0 μmol quanta·m^−2^·s^−1^ and it was significantly lower than in the control stems that were equal to 39.1 μmol quanta·m^−2^·s^−1^ (Figure 5a). Net photosynthesis (Pn) and stomatal conductance of grass pea leaves gradually decreased with increasing NaCl concentrations (Figure 5a), whereas, in stems, Pn not only did not decrease under stress conditions, but increased (threefold) under severe salinity (Figure 5a). Under the same conditions, stomatal conductance of stems did not change in comparison to the control (Figure 5a). Lower Gs resulted in significantly lower transpiration in leaves. Transpiration also decreased in stems under the higher NaCl concentration (Figure 5a). In addition, in the range of light intensity between moderate (100 μmol quanta·m^−2^·s^−1^) and high (2000 μmol quanta·m^−2^·s^−1^) leaf photosynthesis efficiency was significantly lower in plants that were treated with 100 mM NaCl (Figure 5b), while the stem photosynthesis efficiency of the same treated plants was significantly higher in low light intensity (0–50 μmol·m^−2^·s^−1^), and moderate and high light intensity did not differ from control plants (Figure 5b). Conversely, leaf photosynthesis efficiency of seedlings from 50 mM NaCl did not change, but the efficiency of stem photosynthesis in the same conditions was significantly lower than in the control (Figure 5b).

**Figure 6:**
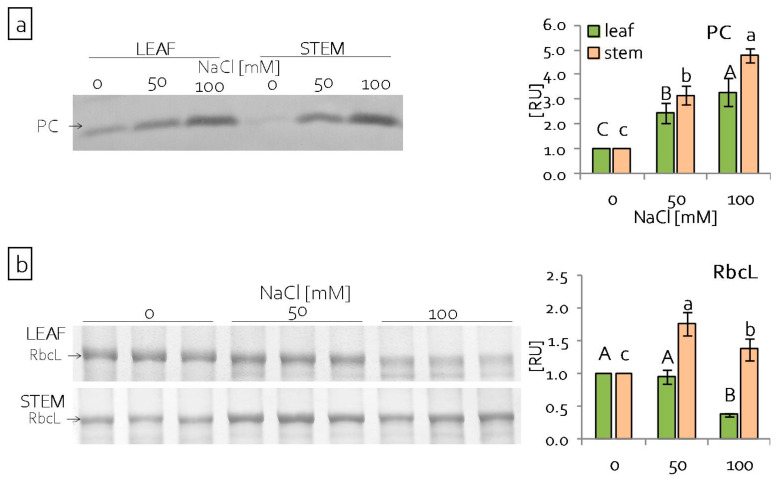
Content of (**a**) plastocyanin (PC) and (**b**) large subunit of RuBisCo (RbcL) of grass pea leaf and stem photosynthetic apparatus under salinity; content of proteins expressed as relative units [RU]; different letters—statistically significant differences within each organ (leaf-uppercase, stem-lowercase) at *p* ≤ 0.05; (*n* = 3).

The quantitative participation of Plastocyanin (PC) and an enzyme of the Calvin-Benson cycle (RuBisCo) were estimated by SDS-PAGE and immunoblotting in order to examine the effect of salinity on particular protein elements of the photosynthetic apparatus. PC, a small protein and long-range electron carrier between PSII and PSI [45], increased gradually with the increasing concentration of NaCl in both the leaves and stems of grass pea seedlings (Figure 6a). However, the increase of PC under 100 mM NaCl was threefold in leaves and almost fivefold in stems, as compared to organs in control conditions (Figure 6a). We also determined the content of RuBisCO, the most important enzyme in carbon fixation of plants [46]. The content of RbcL (RuBisCo large subunit) decreased in leaves under severe salinity (Figure 6b). Conversely, in stems, the RbcL content increased both under 50 and 100 mM NaCl in comparison to the control (Figure 6b).

### 2.4. Reorganisation of the Leaf and Stem Photosynthetic Apparatus under Salinity Stress

**Figure 7:**
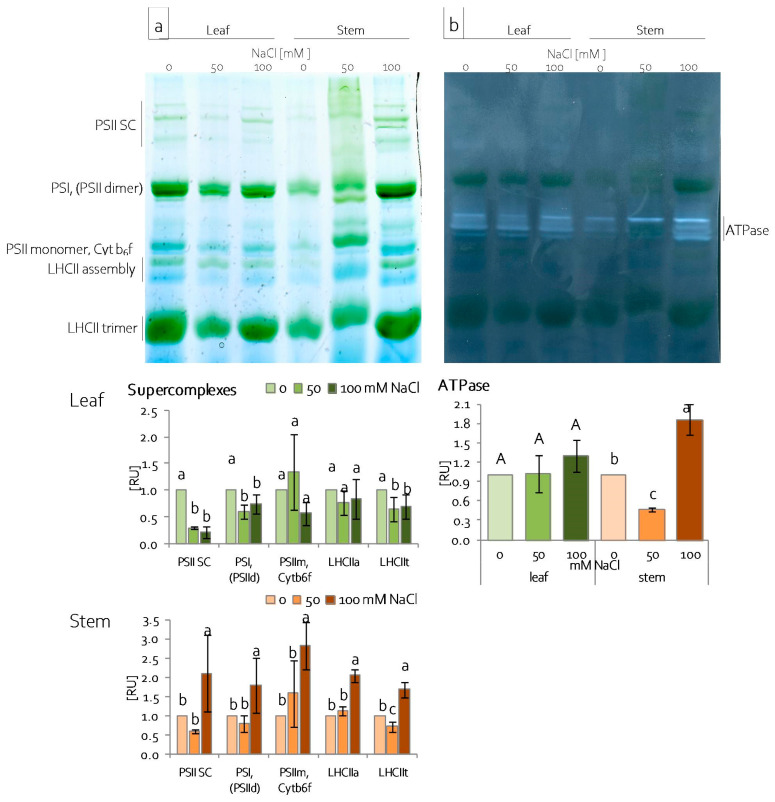
Protein complex and supercomplex content (**a**) and ATPase activity (**b**) in leaf and stem chloroplasts under salinity; PSII—photosystem II, SC—supercomplex, PSIId—PSII dimer, PSI—photosystem I, PSIIm—PSII monomer, *Cytb6f*—cytochrome *b6f*, LHCIIa—LHCII assembly, LHCIIt—LHCII trimer; content of proteins expressed as relative units [RU]; different letters—statistically significant differences within each organ (leaf-uppercase, stem-lowercase) at *p* ≤ 0.05; (*n* = 3).

Changes in protein composition of the photosynthetic apparatus were determined in chloroplasts, which were isolated from leaves and stems of grass pea seedlings by BN-PAGE electrophoresis and second dimension electrophoresis. BN-PAGE electrophoresis allowed for the fractionation of chloroplast protein complexes based on their size and shape. The presence of Serva Blue G250 allowed for the visualisation of the most abundant protein complexes and supercomplexes, i.e., PSI, PSII, LHCII (light harvesting complexes of PSII), *Cytb_6_f,* and ATPase. Depending on the Chl content, the bands were blue or blue-green coloured (Figure 7a). Salinity affected the number of protein complexes and supercomplexes. Generally, in leaves, the PSII supercomplex and PSI complex, as well as the LHCII trimer, decreased under both of the NaCl concentrations tested (Figure 7a). A higher NaCl concentration led to the increase of all the identified complexes and supercomplexes in grass pea stems (Figure 7a). The in-gel activity assay for ATPase activity revealed activity zones that are visible in the middle between the PSIId|PSI and PSII monomer|*Cytb_6_f* suprecomplexes (Figure 7b). Depending on the organ examined and treatment, a variable number and different intensity of activity zones were noted. In leaf chloroplasts, four activity zones were recorded, but the salinity did not significantly affect the total ATPase activity (Figure 7b). In stem chloroplasts, four activity zones were only visible under the higher NaCl concentration (Figure 7b). Additionally, severe salinity (100 mM NaCl) significantly increased, and moderate salinity (50 mM NaCl) significantly decreased the total ATPase activity (Figure 7b).

**Figure 8:**
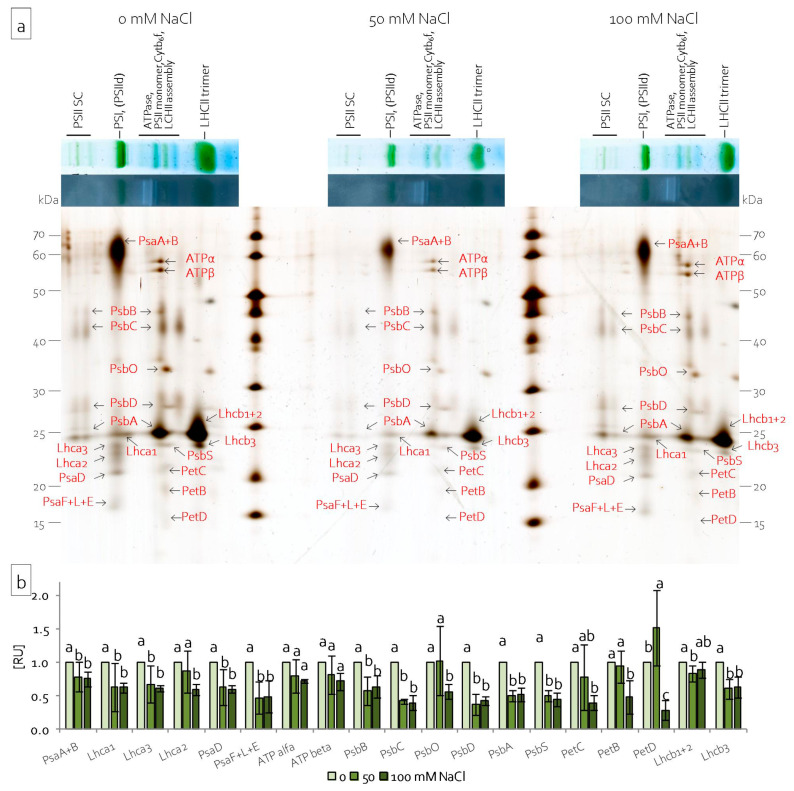
Protein composition of grass pea leaf chloroplasts under salinity; (**a**) two-dimensional protein patterns; (**b**) content of particular proteins; content of proteins expressed as relative units [RU]; different letters—statistically significant differences within each protein at *p* ≤ 0.05; (*n* = 3).

**Figure 9:**
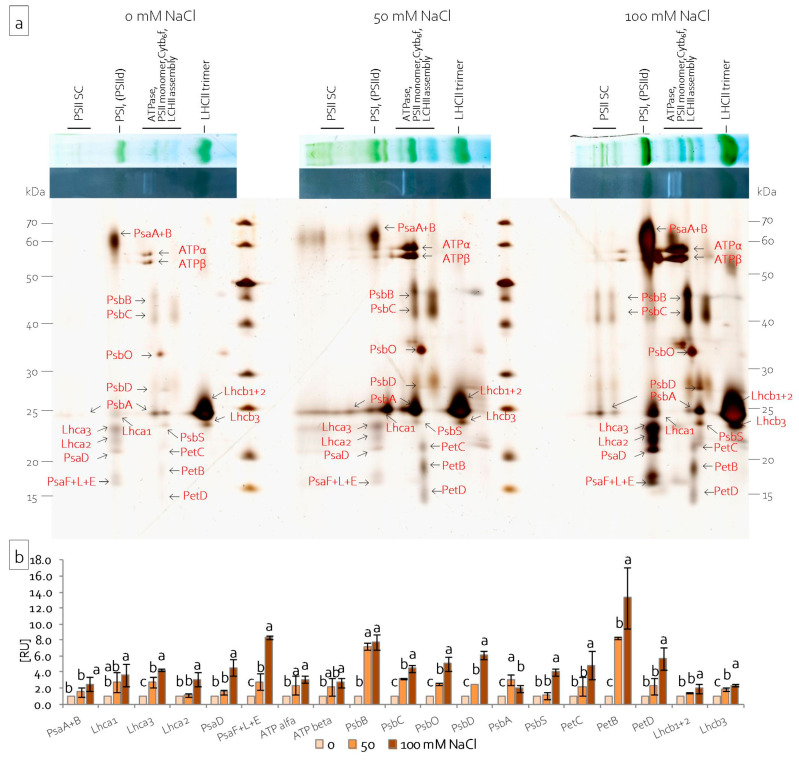
Protein composition of grass pea stem chloroplasts under salinity; (**a**) two-dimensional protein patterns; (**b**) content of particular proteins; content of proteins expressed as relative units [RU]; different letters—statistically significant differences within each protein at *p* ≤ 0.05; (*n* = 3).

Single lanes of BN gels after the in-gel activity assay were denatured and separated in the second dimension in the presence of SDS in order to analyse the subunit composition of chloroplast complexes. Analysis of the obtained two-dimensional protein pattern allowed for the identification of the protein complexes by their subunit composition. Characteristic protein patterns suggested which bands corresponded to which complexes and supercomplexes [47,48]. The same protein pattern was observed for both leaf and stem chloroplasts (Figure 8a and Figure 9a). The first green-coloured BN band was resolved into nine proteins. The largest protein spot represented PsaA and PsaB. The three proteins of apparent molecular weight between 22 and 25 kDa represented subunits of the light harvesting complex: Lhca1, Lhca2, and Lhca3. The two lowest protein spots (between 17 and 20 kDa) represented the subunits of PSI: PsaE, PsaF, PsaL, and PsaD. The group of blue and green bands corresponding to supercomplexes of ATPase, PSII, *Cytb_6_f,* and LHCII (Figure 7, Figure 8 and Figure 9) resolved into 11 proteins (Figure 8a and Figure 9a). the ATPase complex resolved into two spots of 55 and 54 kDa, which were identified as ATPα and ATPβ subunits (Figure 8a and Figure 9a). Lower spots were identified as subunits of PSII: PsbB, PsbC, PsbO, PsbD, PsbA, and PsbS. Some of these proteins were also visible under groups of BN bands, corresponding to the PSII supercomplex (Figure 8a and Figure 9a). The lowest were PetB, PetC, and PetD proteins building the *Cytb_6_f* complex (Figure 8a and Figure 9a). The largest green-coloured BN band separated into two spots. The intense spot of 24 kDa corresponded to subunits Lhcb1 and Lhcb2. The third visible subunit of the LHCII complex formed a less intense spot that was just below the previous one and represented the Lhcb3 protein (Figure 8a and Figure 9a). Densitometric analysis of the obtained gels also revealed changes in the protein content with regard to salinity intensity (Figure 8b and Figure 9b). Salinity decreased the content of PSI proteins PsaA, PsaB, PsaF, PsaL, and PsaE and LHCI proteins Lhca1, Lhca2, and Lhca3 in the leaf chloroplasts (Figure 8b). Moreover, it also decreased the content of PsbA, PsbC, PsbD, and PsbS, proteins belonging to the PSII complex, and the content of Lhcb1, Lhcb2, and Lhcb3 proteins, which build the LHCII complex (Figure 8b). Severe salinity decreased the content of subunits of the *Cytb_6_f* complex, but it did not change the content of the ATPase complex subunits (Figure 8b). However, in stem chloroplasts, severe salinity (100 mM NaCl) increased the content of all the identified proteins (Figure 9b).

**Figure 10:**
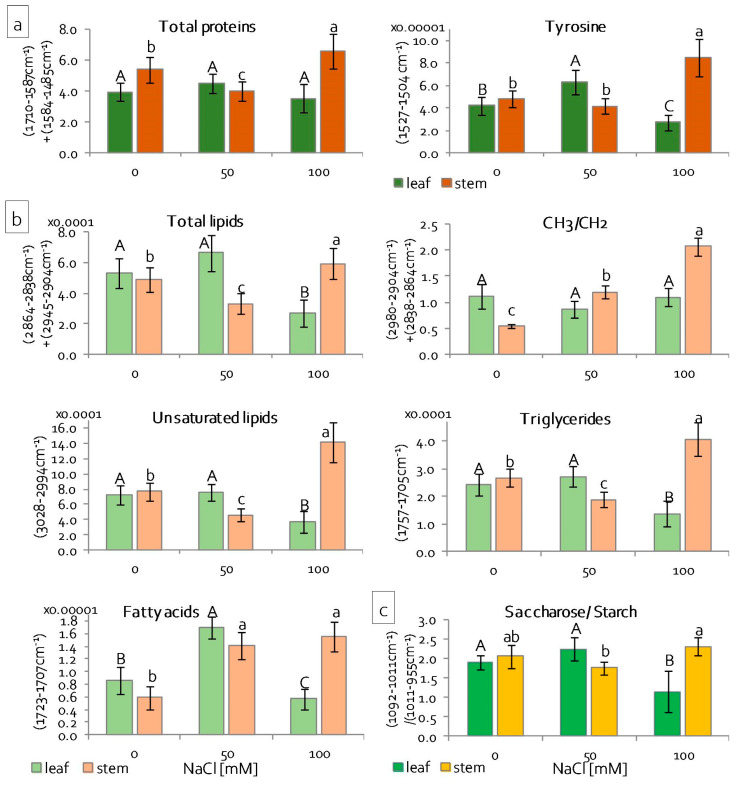
Semi-quantification of chloroplast biocomponents in leaves and stems identified in attenuated total reflectance—Fourier-transform infrared (ATR-FTIR) spectra: (**a**) proteins and Tyr residues, (**b**) lipid fractions, and (**c**) saccharides. Intergraded spectral regions are listed in Experimental Section; different letters—statistically significant differences within each organ (leaf-uppercase, stem-lowercase) at *p* ≤ 0.05; (*n* = 3).

The absorbance of most plant organics existed in the mid-infrared region (4000–900 cm^−1^), so ATR-FTIR (attenuated total reflectance—Fourier-transform infrared) spectra of stem and leaf chloroplasts were recorded in order to examine the effect of salinity on lipids, proteins, and sugars. This method of chemical sensing only required a few milligrams of samples and it was label-free. The rationale of such an experiment was based on the expectation that spectral features should result from metabolism alternation in chloroplast membranes, protein patterns, and poly/saccharides. Raw spectra, their second derivatives, and a band assignment to biocomponents are summarised in the Supplementary materials (Table S2 and Figure S2). The ATR-FTIR spectra clearly showed that some differences of intensities, shapes, and peak positions of the spectra appeared in the entire spectral region, especially under severe salinity conditions. The differences were instinctively pronounced in the calculated integral intensities of bands assigned lipid, protein, and sugar fractions, as presented in Figure 10. These values were used in order to estimate their contents.

The graphs in Figure 10a showed the content of proteins and tyrosine residues calculated from absorbances of amide I and II bands, and *a* band at 1516 cm^−1^, respectively (Table S2 and Figure 9b) [49]. The overall content of all proteins and tyrosine residues escalated significantly in stems under 100 mM NaCl due to the increase of salinity. While the leaf chloroplasts did not change the protein metabolism, the Tyr level increased at 50 mM NaCl and then decreased at the higher concentration of the salt (Figure 10a). As can be seen in the region of amide I and II bands (S2c), stem chloroplasts that were treated with the high concentration of NaCl were characterised by a higher intensity of the spectral bands with the maxima at 1633 and 1541 cm^−1^. These signals can be assigned to β-sheet conformations, suggesting the process of protein aggregation [49,50]. Statistical analysis was carried out in the main lipid region (3050–2800 cm^−1^), which contained signatures of CH_2_, CH_3_, and =CH moieties in the acyl chains, as well as bands of the C=O groups in triacylglycerols and free fatty acids (1750–1700 cm^−1^), in order to assess the similarities and dissimilarities among the membranes of chloroplasts and their response to salinity (Figure S2 and Figure 10b, Table S2) [49,50,51]. A total lipid content in membranes of the leaf chloroplasts slightly increased at 50 mM NaCl, and the membranes lost their lipid fraction due to the enhancement of the salt effect. The opposite trend was found for stems, which suggested that mechanisms of membrane degradation or protection response are completely different. Next, the ratio of absorbances attributed to the methyl and methylene groups revealed the alternation of molecular structures of the acyl chains in lipids. Its increase suggested the production of lipids with short and/or branched acyl chains due to their fragmentation in the peroxidation process. The corresponding graph in Figure 10b indicated that this process occurred in stems already due to the action of 50 mM NaCl and was enhanced for the high concentration of the salt. Likely, the high production of lipids in the stem chloroplasts was associated with the synthesis of fatty acids with short acyl chains. In leaf chloroplasts, the length of the acyl chain was not altered. Another FTIR parameter of lipid peroxidation was a signal at 3012 cm^−1^, which was attributed to stretches of the olefinic group (=C–H) in fatty acid chains (Figure S2 and Table S2). In this case, the shortening of fatty acid chains in stem chloroplasts was accompanied by the degradation of unsaturated fatty acids in 50 mM NaCl and pronounced synthesis in 100 mM NaCl, similar to changes in the total content of lipids (Figure 10b). Chloroplasts in leaves lost unsaturated lipids only under severe salinity conditions. This observation confirmed the fact that two different processes of membrane transformation must have occurred in both systems. Triglycerides and free fatty acids were detected in the FTIR spectra by absorbances at 1740 and 1716 cm^−1^, respectively. Both of the lipid fractions responded to salinity stress in a different way (Figure 10b). In leaf and stem chloroplasts, the esterification of fatty acids occurred, similar to the changes of the unsaturation degree. Free fatty acids were synthesised at a comparable level in stems, regardless of the concentrations of NaCl. In leaves, they were only produced under mild salinity conditions.

The wide and intense absorbance in the 900–1200 cm^−1^ region belonging to the stretching vibrations of the C–O and C–C groups was an indication that the chloroplast from stems and leaves contained a mixture of poly/saccharides [52,53]. According to the reference FTIR spectra of numerous saccharides, we assigned bands at 1048 and 990 cm^−1^ to saccharose and starch, respectively, with some contribution of monosaccharides (Figure S2 and Table S2). The second derivative FTIR spectra showed the alternation of these bands’ intensities, which suggested that salinity induced the transformation between starch and saccharose, as illustrated by a graph in Figure 10c. The saccharide/starch ratio was altered differently, which indicated that transformation between both sugars depended on the localisation of chloroplasts, as well as salt concentration.

## 3. Discussion

From the beginning of salinity emergence, the plants were subjected to two types of stress: osmotic and ionic. The distinction between the responses induced in plants by these two types of stress was partly possible, because they were staggered over time (at least in the initial stages) [4]. The growth rate of root and shoot cells was relatively quickly inhibited by osmotic stress. Conversely, the effects of ionic stress appeared, depending on the rate of accumulation of ions in the plant, within a few hours or from several to a dozen days from the moment of salt appearance in the soil solution [54,55]. The tolerance to salinity stress occurs when the plant has mechanisms that make it tolerant to both ‘component’ stresses. If the plant has the mechanisms that are responsible for tolerance to osmotic stress, but is not tolerant to ionic stress, it may be characterised by good growth parameters in the initial stage of salinity stress. However, after salt ions reach a toxic concentration in the cells, its growth and development will be inhibited [54]. In the opposite case, the plant’s tolerance to osmotic stress will constantly limit the growth and development. Only the combination of tolerance to osmotic and ionic stress allows for optimal growth and development under salt stress conditions throughout ontogenesis [4].

In order to cope with ionic stress, plants can limit the entry of harmful ions to roots and their further transport to shoots or after uptake sequester them efficiently in vacuoles to minimise their concentration in the cytoplasm [4,7,14,56]. Detailed analysis showed that the distribution of Na accumulation was different in leaves and stems (Figure 2a). In the leaves, the Na^+^ content increased with increasing NaCl concentration, but, in stems, Na accumulation was on the same level, regardless of NaCl concentration. This may suggest a mechanism that effectively moves sodium ions from stems to leaves under 100 mM NaCl, which allows for stems to function, but leads to leaf death. It is assumed that, if cells can function normally despite high concentrations of harmful ions, they are effectively sequestered in the vacuoles [4]. Grass pea seedlings under 50 mM NaCl, despite increasing content of Na^+^ ions, retained the ability to grow and develop, which may indicate effective Na^+^ sequestration in the vacuoles. Additional to Na^+^ sequestration, plants can endure ion toxicity by taking up and accumulating potassium [57]. A higher K^+^ ion uptake was observed in salinity tolerant plants than in salinity sensitive plants [57,58]. Thus, an increased accumulation of K^+^ can be an important indicator of plant acclimatisation to salt stress. Conversely, the uptake of K^+^ ions, which form a counterbalance to Na^+^ ions, becomes extremely energy-expansive for plants under salinity stress conditions and it could be replaced by other mechanisms [59,60]. In grass pea seedlings, a gradual decrease of K^+^ accumulation in stems under increasing NaCl concentration was observed, which could be a result of such mechanisms.

The translocation of harmful ions to grass pea shoots suggests that the shoots’ response to stress is essential in the acclimatisation of plants to stressful conditions. Moreover, a differential response was observed between the leaves and stems of seedlings. Our results, showing the condition of the plasma membranes (MDA content) in plants under 100 mM NaCl (Figure 2b), indicated that the leaves were exposed to greater stress than the stems. The observations of ion distribution in the aerial parts of seedlings (Figure 2a) and seedling morphology (Figure 1b) confirmed this. These results show that NaCl at a 100 mM concentration, while not lethal for seedlings, as evidenced by both the morphological and physiological condition of the stems, is highly toxic and it generates severe stress.

Stress tolerance results from the ability to maintain the structural integrity of the cell. In the first place, it depends on the stability of biological membranes, both in cells and cell organelles. The stability of biological membranes is determined by their composition, in particular the content of unsaturated fatty acids that are included in membrane phospholipids [61]. The main unsaturated fatty acids in membranes are 16- and 18-carbon ω-3 trienoic acids (i.e., 16:3 and 18:3 fatty acid), and alterations in their level determine the fluidity of biological membranes [62,63]. In contrast, changes in the degree of unsaturation (i.e., membrane fluidity) constitute the main element of acclimatisation to stress factors [61]. A high degree of membrane fatty acid unsaturation allows for high fluidity of the membranes and, thus, mitigates the harmful effects of the stressor. Stress conditions significantly modify the activity of key cell organelles, of which chloroplasts are the most important, both in terms of structural and functional integrity. The stability of their action is related to proper fluidity of the thylakoid membranes. In turn, the number of chloroplasts in the cell, as well as the number of thylakoids in the chloroplast (chloroplast ultrastructure), imply the cell’s acclimatisation ability [61]. Under stressful conditions, the fluidity of thylakoid membranes is maintained by the reduction of their number in the chloroplast. This reduces the risk of uncontrolled generation of ROS [64,65]. Many authors observed a decrease in the degree of unsaturation of fatty acids in biological membranes, because of various stresses, such as low and high temperature, salinity, drought, nitrogen deficiency, and the presence of heavy metals [61]. In grass pea, two completely different strategies were observed for the acclimatisation of thylakoids to salinity stress. The leaves showed a decrease in lipid content, the degree of their unsaturation, and a decrease in the content of triacylglycerols (Figure 10b). At the same time, the length of the fatty acids did not change. The observed changes indicated a significant reduction of the thylakoid number and a decrease of their fluidity. In stems, the higher salt concentration increased the content of lipids, triacylglycerols, and unsaturated fatty acids (Figure 10b). At the same time, a shortening or branching of the hydrocarbon chains of fatty acids included in the lipids was observed. Such changes may indicate an increase in the fluidity and integrity of the membranes that build chloroplasts, which is also evidenced by the lower dynamics of lipid peroxidation in relation to the leaves (Figure 2b). In addition to changes in lipid content, stress factors also modify protein metabolism, which leads to changes of their amount and structure. Akyuz et al. [66] observed a decrease in the protein content in soybean mutants tolerant to salinity, including a decrease in the content of alpha helix structures, while increasing the content of the beta-sheet, as well as beta-turn structures, under high salinity conditions as compared to the control. Conversely, in the halophyte *Sesuvium portulacastrum*, salt stress caused a significant increase in the protein content, in both the roots and leaves [52]. Our results revealed no changes in the protein content in the leaves of grass pea seedlings, regardless of NaCl concentration (Figure 10a). In turn, in the stems, the higher salt concentration increased the protein content, especially proteins with the beta-sheet conformation (Figure 10a and Figure S2c). Proper functioning of proteins is associated with the maintenance of their spatial structure. Stress factors disturb this structure, both at the stage of protein formation and in its active form [66]. Plants have developed many mechanisms for protecting the spatial structure of proteins under stressful conditions, including the accumulation of proline, whose main function is to stabilise the tertiary and quaternary structure of proteins, especially enzymatic ones [67]. Studies on salinity stress tolerant soybean mutants showed a high correlation between proline content and high protein content in the beta-turn conformation, which resulted in a high resistance to salt stress [66]. Increased proline accumulation was also observed for grass pea stems under 100 mM NaCl (Figure 2f). At the same time, there were different metabolic responses in different organs. While the high content of proline in the leaves was not related to efficient functioning of the photosynthetic apparatus, in the stems, the photosynthetic apparatus was characterised by high efficiency (Figure 4c). Apart from stabilising the spatial structure of proteins, proline often appears in cells as an osmolyte, participating in osmotic adjustment. Osmotic adjustment most often consists of the formation and accumulation of organic compounds as a counterbalance to NaCl that is accumulated in toxic concentrations in the vacuole [59,68,69]. Other compounds, except proline, whose primary function is osmotic action, are soluble sugars [70,71]. In the presented research, an increase of soluble sugar content in stems under 100 mM NaCl was observed, which was confirmed by both FT-IR and spectrophotometric analysis. In addition, there was also an increase in the accumulation of ODAP (Figure 2e), a compound characteristic for this species, which is believed to be an osmoregulator and osmoprotectant [8,72]. Moreover, in stems under 100 mM NaCl, the rapidly increasing content of ODAP and soluble sugars, which was not accompanied by further accumulation of sodium, may indicate an effective mechanism of acclimatisation of these organs to high salinity.

Photosynthesis is a process that directly or indirectly determines life on Earth [73]. Thus, an efficient and productive photosynthetic apparatus is *sine qua non* for the life of plants. It consists of three main protein–pigment complexes that are located in the thylakoid membranes, PSII, cytochrome *b6f*, and PSI, as well as enzymatic proteins that are embedded in the chloroplast stroma [74]. PSII is composed of three supercomplexes: (1) LHCII—responsible for the perception and transmission of photosynthetic active radiation, (2) PSII RC, where primary charge separation and stabilisation of captured solar energy occurs [75], and (3) OEC [74]. LHCII contains 70–85% of total Chl, as well as Car pigments, among which lutein and components of the xanthophyll cycle (violaxanthin, anteroxanthin, and zeaxanthin) play major roles in dissipating the excessive amount of absorbed energy, protecting the photosynthetic apparatus against PSII oxidation that could lead to photoinhibition [76,77]. One of the effective strategies for reducing the induction of ROS during salinity stress is the limitation of PAR conversion by reducing the size of photosynthetic antennas [78]. The reduction is achieved by the limitation of Chl synthesis [79], LHCII structural proteins synthesis [80], and/or synthesis of Chl docking proteins in LHCII antennas [81].

Salt stress leads to disturbances in the synthesis pathway of Chls [8,82]. Na^+^ ions, by disrupting the activity of 5-aminolevulinic acid (ALA) dehydrogenase, impair the synthesis of ALA, which is a precursor of porphyrins and, thus, Chls [82]. Moreover, the expression of genes related to porphyrin biosynthesis may also be changed [83]. Santos [82] showed that mild salt stress may also increase the activity of chlorophyllase, contributing to accelerated degradation of Chl. As a result of these disorders, a decrease of photosynthetic pigments content was noted in plants that were exposed to NaCl [8,35]. It can be assumed that in the presented research, the decrease in pigment content was due to impaired Chl *a* synthesis, as well as the rapid degradation of Chl *a* forming the PSII RC. Additionally, the content of Car decreased (Figure 4a), which may be due to either (1) an impairment of their synthesis pathway [84], (2) their enzymatic oxidation by carotenoid cleavage dioxygenases (CCDs) [85], or (3) non-enzymatic oxidation by ROS [86].

LHCII constitutes two types of antennas: external antennas that formed by the trimers of Lhcb1, Lhcb2, and Lhcb3, and internal antennas, which are directly adjacent to the core part of PSII, i.e., monomeric proteins Lhcb4 (CP29), Lhcb5 (CP24), and Lhcb6 (CP24) [77,87]. The excess of Na^+^ and Cl^–^ ions disrupts the synthesis of antenna proteins, especially external antennas, which results in reducing the number of supercomplexes that are composed of two protein trimers (Lhcb1-Lhcb2-Lhcb3) [88]. These structures are in the granal area of thylakoids and, with the addition of effective utilisation of PAR radiation, they are responsible for maintaining the appropriate architecture of chloroplasts [87,89]. The toxic effect of NaCl leads to the reduction of both the size and efficiency of LHCII antennas, as well as the decrease in the linear electron transport (LET) rate [77]. In the leaves of examined grass pea seedlings, the content of Lhcb1, 2, and 3 proteins decreased, regardless of the intensity of salinity (Figure 8a,b). In the stems, the content of these proteins not only did not decrease, but increased, under severe salt stress (100 mM NaCl) (Figure 9a,b), which indicated the correct structure of chloroplasts allowing for potentially efficient electron transport. As a result of the observed changes in the leaves, the area of the antennas decreased with simultaneously more effective absorption of radiation both per cross section of chloroplast (TRo/CSo) and a single active RC (TRo/RC). Conversely, in the stems, the increased efficiency of energy trapping (TRo/CSo) resulted from the increased size of the antennas (Figure 4c).

In the leaves, the decrease in antenna number was also accompanied by an increase in the number of closed RCs (Vj). Similarly, in the stems, a decrease in active RCs was observed, but only under 100 mM NaCl. Huang et al. [90] reported that disturbances in the function of PSII RC may result from the inhibition of the synthesis of key proteins that form the structure. Additionally, Shu et al. [88] observed, in *Cucumis sativus*, that salt stress downregulated PsbA and PsbB proteins, as well as diminished Q_B_, while internal antennas (CP24 and PsbC), external antennas (LHCII-trimmer), and PsbD proteins were upregulated. However, it seems that, apart from the obvious changes in the amount of newly synthesised proteins of the PSII RC complex, salinity disturbs its function through changes in protein tertiary and quaternary structures [91]. A decrease in the amount of *de novo* synthesised core proteins of the PSII RC (PsbA and PsbD), as well as the internal antenna protein (PsbC), was observed in the leaves of grass pea (Figure 8a,b). Conversely, in grass pea stems, the amount of key PSII elements increased under stress conditions (Figure 9a,b).

Abiotic stress, which includes salt stress, leads to disturbances in the structure and function of the OEC, interacting with the PsbC protein [41,92,93]. Damage to the OEC leads to disturbances in water splitting, which limits the amount of electrons transferred to the PSII RC. As a result, the prolonged oxidised state of PSII is observed, which is lethal to its surroundings, leading to photooxidation and then photo-inhibition [78,93]. The water-decomposing complex is made of three proteins (PsbO, PsbP, PsbQ), which stabilise the functional manganese cluster (Mn_4_CaO_5_) and optimise the level of cofactors (Ca^2+^ and Cl^−^) [93,94]. Under salt stress, in plant’ cultivars resistant to salinity, as well as in halophytes, an increase of PsbO protein synthesis was observed, which reduces the adverse effects of the stressor on the photosynthetic apparatus [78,95]. In the leaves of grass pea, salt stress did not induce an increase in PsbO synthesis (Figure 8a,b). Consequently, a reduction in the efficiency of the OEC was observed (Fv/Fo), leading to a limitation of PSII activity on the donor side (Figure 4c). In turn, in the stems, strong salt stress induced PsbO synthesis; hence, no decrease in OEC efficiency was observed (no limitation on the donor side of PSII RC) (Figure 9a,b).

In contrast, salt stress caused limitations on the acceptor side of PSII in both leaves and stems. This limitation was due to disturbances in electron transport in the immediate vicinity of the RC between Q_A_ and Q_B_ (φ_Eo_) (Figure 4c). The disruption of electron transport between Q_A_ and Q_B_ resulted in the blockage of linear electron transport (LET), increasing the risk of PSII inactivation due to the appearance of the chlorophyll triplet state [96]. It was counteracted by a single carotene molecule that was present in the PSII RC, as well as zeaxanthin located in photosynthetic antennas. These Car molecules intercepted an electron from the chlorophyll triplet state, dissipating its energy in the form of heat [97]. In grass pea leaves, an increase in the dissipation of excess absorbed energy in the form of heat was observed both per cross-section (DIo/CSo), reflecting the activity of the xanthophyll cycle, and per single RC (DIo/RC), reflecting carotene activity in the PSII RC. In the stems, no change in the amount of energy that is dissipated per single RC (DIo/RC) confirmed the lack of limitation on the PSII RC donor side (Figure 4c), lowering the danger of uncontrolled oxygen reduction and ROS generation.

In order to protect themselves against excessive, uncontrolled generation of reactive oxygen species, the plants increase the activity of enzymes, including catalase and ascorbate peroxidase [98]. Because catalase has a higher reactivity and lower affinity to H_2_O_2_, it is more strongly involved in scavenging free radicals than in the signalling role [98,99]. An increase in the catalase content was also observed in grass pea leaves and stems, as in many plants under saline conditions [98,100] (Figure 3b). While CAT mainly occurs in peroxisomes [101], ascorbate peroxidase occurs in at least four cell compartments (constituting different isoforms): in the stroma (sAPX) and thylakoids (tAPX) of chloroplasts, peroxisomes (pAPX), and cytosol (cAPX) [102]. Chloroplastic isoforms of APX are involved in the photosynthesis process under stress conditions and they both participate in the water-water cycle, during which water is reduced in the Mehler reaction [103,104]. It follows that chloroplastic APXs are essential for photoprotection, but sAPX additionally plays a key role in the response to oxidative stress in the early development stage [103]. In grass pea seedlings, the content of thylakoid and stromal APX isoforms decreased in the leaves under 100 mM NaCl, which indicated that the production of H_2_O_2_ in these organs exceeded the threshold level [105]. Conversely, under the same conditions, no changes in the amount of the stromal APX isoform were noted in the stems, which may suggest some mechanisms that are involved in preventing the production of H_2_O_2_ (evidence that the level of H_2_O_2_ in these organs was lower, suggesting an effective mechanism preventing its formation). Additionally, an increase in the cytosolic APX content in the stems was noted under 100 mM NaCl, while, in the leaves, it was significantly reduced (Figure 3a). It is believed that cytosolic APX can protect chloroplastic APXs, and its presence is crucial in the removal of H_2_O_2_ that is produced during the photosynthesis process [103,106].

The disruptions of LET also imply disorders in the formation of a proton gradient between stroma and lumen, leading to a reduction of ATP synthesis, finally reducing the rate of photosynthesis [74]. Such disturbances were observed in plants that were growing under Ni and Cd stress [107], as well as in the presence of excess NO_2_^–^ [108] or NaCl [109,110]. Non-heme Fe is involved in the electron transport between Q_A_ and Q_B_. On the one hand, it maintains the appropriate spatial structure between Q_A_ and Q_B_; on the other hand, it binds the HCO_3_^–^ ion and, thus, participates in the protonation of Q_B_^2−^ [74]. Zhang et al. [108] believed that the NO_2_^–^ anion binds to non-heme iron instead of the HCO_3_^–^ ion, which inhibits electron transport between Q_A_ and Q_B_.

The disruption of LET may also result from limitations in the amount and movement speed of electron carriers (PQ and PC), limitations in the function of cytochrome *b6f*, and damages to PSI, as well as interferences of enzymes that are associated with transported electron usage on the acceptor side of PSI. Differing sensitivity of PC, cytochrome *b6f,* and PSI to salt stress was observed [110,111,112,113]. In grass pea leaves, no change in the plastoquinone pool (Area) (Figure 4c), an increase of PC content, and a reduction of cytochrome *b6f* (PetB, PetC, and PetD proteins) amount (Figure 8a,b) was observed (Figure 6a). Moreover, PSII, PSI activity, and energy flow between them decreased (PI_Total_) (Figure 4c). In addition, salt stress reduced the size of external antennas that are associated with PSI, i.e., Lhca1 protein, as well as the number of proteins that form the PSI RC (PsaA and PsaB proteins) (Figure 8a,b). Such construction provides the potential risk of long-term oxidation of PSI, especially when a strong limitation in the number of electrons transported from PSII occurs [114]. PSI can remain in an oxidised state for longer periods than PSII without serious damage to the membranes. However, the prolonged state of its oxidation leads to irreversible structural damage to the thylakoid membrane, as well as degradation of the PSI RC itself [114]. Additionally, in grass pea leaves under 100 mM NaCl, a decrease in the quantum efficiency of the transported electron reduction by the ferrodoxine-dependent system on the acceptor side of PSI was observed. However, this parameter does not consider the photochemical efficiency and activity of PSI and its immediate surroundings [115,116]. Thus, a decrease in the value of this parameter may indicate both disturbances in transport within PSI, as well as dysfunction of ferrodoxin and its dependent reactions, which will ultimately limit the linear and alternative pathways of electron transport. Alternative electron transport pathways include both the water–water cycle [117], transport while using the PGR5-PGRL1 complex, and NDH and NDH, together with PTOX (chlororespiration) [118]. They lower the risk of overreduction of PSII, which leads to the production of ROS [111]. In plants with PSI donor limitations, alternative electron transport pathways primarily include the PGR5-PGRL1 complex pathway, preventing P700 oxidation [119]. In contrast, plants that are subjected to salinity stress follow the NDH-dependent pathway due to additional energy requirements [120]. In grass pea leaves under higher NaCl concentrations, due to the limitations on the acceptor side of PSII, the danger of overreduction of PSII is close to zero. Thus, no activity on the water-water cycle is to be expected. However, the cycle with the use of NDH seems to be more likely.

In grass pea stems, under salinity conditions, the plastoquinone pool (Area) (Figure 4c) and PC content (Figure 6a) increased. Moreover, the content of cytochrome *b6f* (Pet C, PetB, PetD proteins), as well as the PSI RC (PsaA and PsaB proteins) and external and internal antennas (Lhca1, Lhca2 and Lhca3 proteins), increased (Figure 9a,b). The increasing amount of cytochrome and PSI building proteins with limited availability of electrons from PSII may also generate a danger of PSI oxidation [121]. In order to reduce the risk of photooxidation in plants, an alternative electron transport pathway is activated while using the ferrodoxin-dependent thioredoxin system, which allows for safe electron circulation around PSI as part of cyclic electron transport (CET), generating a proton gradient that is used by ATP synthase for ATP synthesis [122]. Additionally, by acidifying the lumen with the PsbS protein, CET activates the dissipation of excess energy (NPQ—non-photochemical quenching), protecting PSII against photo-oxidation [122]. Second dimension electrophoresis showed the presence of complexes comprising cyt *b6f*, LHCI, and PSI and ATP synthase, which confirms the presence of CET in grass pea stems. The functionality of the complex that formed in this way was confirmed by ATPase activity analyses (Figure 7b). ATPase activity was the highest in the so-formed complex in stems under 100 mM NaCl. The abundance of the PSI, LHCI, and ATP synthase complex indicates a reduced number of granal thylakoids and a predominance of stromal thylakoids, because ATP synthase is closely related to the unpackaged areas of the thylakoids [123,124]. Additionally, this complex also included the Lhcb1+2 proteins. The presence of such a complex may result from the phosphorylation of LHCII antennas in the membranes of stromal thylakoid and/or in the periphery of the granal thylakoids and their attachment to the abundant LHCI-PSI complexes there [125]. However, further research is required in order to confirm such a phenomenon.

Salinity implies disturbances in the Calvin–Benson cycle function, including significant limitations in the number and activity of enzymes that are involved in carboxylation, mainly RuBisCo [104]. The decrease in RuBisCo content in response to salinity stress has been observed in many plant species, which was mainly associated with disruptions of large RuBisCo subunit (RbcL) synthesis [126]. At the same time, through the osmotic effect, salt stress limits the carboxylation rate in many species by inducing the closure of the stomata and reducing the availability of CO_2_ [127]. Conversely, some authors, in conditions of limited access of CO_2_, observed an increase in RuBisCo content in response to moderate salt stress, explaining this by the induction of photorespiration as a mechanism protecting PSII against photooxidation [128,129]. In grass pea leaves, a decrease in RbcL content was observed under 100 mM NaCl (Figure 6b), while the decrease in stomatal conductance had already occurred under moderate salt stress (50 mM NaCl) and intensified under severe salt stress (100 mM NaCl) (Figure 5a). In turn, the RbcL content increased in the stems, regardless of the NaCl concentration (Figure 6b), whereas the stomatal conductivity decreased under moderate salinity, but it did not change under severe salinity (Figure 5a). The observed changes in the amount of RbcL and stomatal conductance in the stems in combination with the reduced antenna system, as well as the limitation in electron transport between Q_A_ and Q_B_, minimise the probability of photorespiration.

As a result of changes that are related to photosynthetic apparatus reconstruction under salt stress, both in the leaves and stems of grass pea seedlings, the efficiency of CO_2_ carboxylation changed (Figure 5b). The intensity of photosynthesis in the leaves that were subjected to moderate salt stress did not change when compared to the control, indicating effective acclimatisation mechanisms. In contrast, severe salt stress caused a sharp decrease in the efficiency of carboxylation (Figure 5a). Analysis of the light curve and Chl *a* fluorescence indicated limitations in the number of transported electrons in linear electron transport. However, a sharp decrease in stomatal conductivity (*G*s) may indicate a substrate dark phase limitation [128,129]. Consequently, in leaves under 100 mM NaCl, no net photosynthesis (Pn) was observed, but only a reduction in the rate of mitochondrial respiration and photorespiration. Moreover, these results indicated that these organs were not able to perform the photosynthetic function. Rather, they were a place of Na^+^ accumulation. In contrast, plant stems that were subjected to moderate salinity stress showed a slight decrease in the value of net photosynthesis (Pn) under constant light conditions (Figure 5a). In turn, the light curve (Figure 4b), in combination with the parameters of Chl *a* fluorescence (Figure 4c), shows a decrease in the stomatal conductivity value (Figure 5a), and an increase in RbcL content (Figure 6b), may indicate a reduction in the photosynthesis rate at higher radiation intensities, resulting from photorespiration [128,129]. Plant stems exposed to strong salinity stress were characterised by an increase in photosynthesis intensity at constant light intensity (Figure 5a). Simultaneously, a detailed analysis of the photochemical reaction phase of photosynthesis (light reactions) indicated a much more effective use of lower radiation intensities and it maintained high photosynthesis efficiency, at the level of control stem photosynthesis efficiency, but at higher light intensities. The stems, both in the control and 100 mM NaCl conditions, showed photoinhibition symptoms at high radiation intensities (Figure 5b). Conversely, the value of the light compensation point (Figure 5a) and the course of photosynthesis at low light intensities (Figure 5b), as well as the photosynthetic apparatus construction of the stems growing under 100 mM NaCl, indicate the reconstruction of the photosynthetic apparatus toward increasing photosynthetic efficiency at lower light intensities.

The phenomenon of stem photosynthesis, even woody ones, is observed in natural conditions, but its efficiency is not as high as in the case of leaves [130,131,132,133]. The results that were obtained in the described research clearly show that, under conditions of severe salt stress, the stems take over photosynthetic function and leaves become the place for harmful ion deposition. Maintaining efficient photosynthesis of the stems enables the plant to generate energy to distribute harmful ions, and it provides assimilates that can be used for continued growth of the plant.

## 4. Materials and Methods

### 4.1. Plant Material, Growth Conditions, and Stress Treatment

Plant material comprised seeds of grass pea (*Lathyrus sativus* L.) cv. ‘Krab’. The seeds were seeded on filter paper in Petri dishes and watered daily with media supplemented with 0, 50, or 100 mM NaCl. The medium was composed of basal macro- and microelements: 7.0 mM N, 1.9 mM P, 7.7 mM K, 4.2 mM Ca, 50 µM Mg, 36 µM Fe, 5.5 µM Mn, 9.3 µM B, 3.1 µM Cu, 2.1 µM Mo, and 1.5 µM Zn. After six days, the seedlings were transferred to a hydroponic system with the respective media. Media (with or without NaCl) were replaced every seven days. The seedlings were cultivated for 25 days at 22 ± 2 °C under white LED lamps (100 μmol (quantum)m^−2^ s^−1^ light intensity) and a photoperiod of 16/8 h (day/night). In each treatment, 30 seedlings were cultivated. The experiment was repeated twice.

### 4.2. Evaluation of Seedling Growth and Conditions under NaCl Stress

#### 4.2.1. Biometric Assessment

After 25 days of cultivation, the length of shoots and roots of each seedling was measured, and the percentage of dry weight content was evaluated in shoots and roots separately. Five plants of each treatment were collected, divided into shoots and roots, weighed, freeze-dried for 48 h, and then weighed again. The percentage of dry weight content was calculated.

#### 4.2.2. Sodium and Potassium Content Determination

Sodium and potassium ion content were analysed in two steps: separately in shoot and root tissues and separately in leaf and stem tissues. Approximately 50 mg of freeze-dried tissue was transferred to open Teflon-coated vessels and digested with 5 mL of 65% HNO_3_ for 10 min and with 2 mL of H_2_O_2_ for 30 min Samples were mineralised for 40 min in a microwave digester (speedwave ENTRY, Berghof, Eningen unter Achalm, Germany). Solutions were filtered, adjusted to 25 mL with Milli-Q^®^ water and analysed using an atomic absorption spectrometer (Thermo iCE3000, Waltham, MA, USA). Calibration curves (Na^+^ and K^+^ standards of trace metal basis purity) were used in order to determine cation concentrations.

#### 4.2.3. Malondialdehyde Content Determination

Malondialdehyde (MDA) content, an indicator of lipid membrane peroxidation level, was determined according to the spectrophotometric methods of Dhindsa et al. [134]. Ten mg of freeze-dried tissue (leaves and stems separately) were homogenised with 0.5 mL of 0.1% trichloroacetic acid (TCA) and then centrifuged (10 min, 15,000 rpm, 4 °C). Extract (0.2 mL) was added to 0.8 mL of 20% TCA containing 0.5% tiobarbituric acid (TBA). The mixture was incubated for 30 min at 95 °C, immediately cooled on ice, and then centrifuged (10 min, 15,000 rpm, 4 °C). The absorbance of the reaction mixture was measured at 532 and 600 nm. The content of MDA was calculated while using the extinction coefficient for MDA ε = 155 mM^−1^ cm^−1^ and the absorbance difference (A532–A600) and expressed in nM MDA g^−1^ s.m.

#### 4.2.4. Antioxidant Capacity Determination

The ferric reducing antioxidant power (FRAP) assay [135] was used in order to determine the antioxidant capacity of grass pea leaves and stems. After homogenisation of 10 mg of freeze-dried tissue in 0.5 mL of 80% methanol, the extract was centrifuged (3 min, 13,000 rpm, room temperature) and mixed with 3 mL FRAP solution and 0.3 mL H_2_O. The FRAP solution consisted of 300 mM acetate buffer (pH 3.6), 20 mM FeCl_3_, and 10 mM tripyridyl-*s*-triazine (TPTZ) in ethanol (10:1:1, *v*:*v*:*v*). After 30 min, the absorbance of the samples was measured at 595 nm while using a UV/Vis Spectrophotometer (JASCO V-530, Artisan Technology Group, Champaign, IL, USA). Standard curves for Trolox (6-hydroxy-2,5,7,8-tetramethylchroman-2-carboxylic acid) were used in order to calculate antioxidant capacity presented as Trolox equivalents.

#### 4.2.5. Sugar Content Determination

The contents of soluble and insoluble sugars were separately determined in leaves and stems of grass pea seedlings while using the anthrone reagent method [136]. To determine soluble sugars, freeze-dried tissue was extracted overnight in 1 mL Milli-Q-ultrapure water (Millipore Direct system Q3). The samples were centrifuged (10 min, 15,000 rpm, room temperature) and supernatants collected. In order to determine insoluble sugars, the pellet after water-extraction, was resuspended in 0.5 mL of 0.1 M H_2_SO_4_ and incubated for 60 min at 80 °C. Anthrone reagent solution (1 g anthrone in 500 mL 72% H_2_SO_4_) was mixed with aqueous or acid extracts and then heated for 15 min at 95 °C. After stopping the reaction on ice, the samples were left to reach room temperature. The absorbance of the samples was measured at 630 nm. A glucose calibration curve was used to calculate the content of sugars (mg g^−1^ dw).

#### 4.2.6. β-N-oxalyl-L-α,β-Diamino Propionic Acid Content Determination

The spectrophotometric method with O-phtalaldehyde reagent (OPT) [137] was used in order to estimate the content of β-N-oxalyl-L-α,β-diamino propionic acid (ODAP) in leaves and stems of grass pea seedlings. After overnight extraction of 10 mg of freeze-dried tissue in 1 mL of 60% methanol, the samples were treated with activated charcoal (15 mg, removal of pigments) and centrifuged (10 min, 15,000 rpm, room temperature). One part of the supernatant was hydrolysed with 3N KOH (30 min, 95 °C). OPT reagent (50 mg OPT, 100 μM β-mercaptoethanol, 0.5 mL 95% ethanol, and 49.5 mL borate buffer) or borate buffer (pH 9.9) was mixed with hydrolysed and unhydrolysed extracts. The mixtures were incubated for 2 h at 38 °C. The absorbance of the samples was measured at 425 nm. The absorbance of each sample was calculated according to the equation: *A* = (*A*_3_ − *A*_4_) − ^1^/_3_(*A*_1_ − *A*_2_), where *A*_1_ is the absorbance of unhydrolysed extract with OPT, *A*_2_ is the absorbance of unhydrolysed extract with borate buffer, *A*_3_ is the absorbance of hydrolysed extract with OPT, and *A*_4_ is the absorbance of hydrolysed extract with borate buffer. The content of ODAP was calculated using the calibration curve made for DL-2,3-diaminopropionic acid (DAP).

#### 4.2.7. Proline Content Determination

The spectrophotometric method with ninhydrin [138] was used in order to determine the proline content in leaves and stems of grass pea seedlings. Freeze-dried tissue (10 mg) was homogenised in 1 mL 3% 5-sulfosalicilic acid dehydrate (C_7_H_6_O_6_S × 2H_2_O) and then centrifuged (15,000 rpm, 10 min, 4 °C). After mixing with acid-ninhydrin and glacial acetic acid, the extract was heated for 1 h at 100 °C. After termination of the reaction on ice, the mixture was extracted with toluene (1 mL). The absorbance of toluene extracts was measured at 520 nm. The proline concentration (mg g^−1^ dw) was determined from a calibration curve that was made for *L*-proline.

### 4.3. Evaluation of Photosynthetic Apparatus Performance under NaCl Stress

#### 4.3.1. Photosynthetic Pigment Content Determination

Photosynthetic pigments were extracted according to the Lichtenthaler [139] method. Approximately 10 mg of freeze-dried tissue (leaves and stems separately) was homogenised with 1 mL of 80% acetone. The samples were centrifuged (15 min, 15,000 rpm, 4 °C). Absorbance of the extract was measured at 663, 646, and 470 nm. The content of Chl *a*, Chl *b*, and Car was calculated according to the Wellburn [140] equations.

#### 4.3.2. Chl a Fluorescence

Changes in the protein composition of the photosynthetic apparatus were determined in chloroplasts, which were isolated from leaves. Chl *a* fluorescence was measured on the leaves and stems of grass pea seedlings while using a Handy PEA spectrofluorometer (Hansatech Instruments, King’s Lynn, UK) with relevant standard procedures. The leaves were dark adapted for 20 min before measurement. The fluorescence was induced by red light of a wavelength λ_max_ = 650 nm and an intensity of 3500 μmol photons∙m^−2^∙s^−1^. Recorded curves were analysed using the fluorometer producer’s soft-ware (PEA-Plus). Selected functional and structural photosynthetic parameters were extracted and calculated according to Jiang et al. [43], Kalaji et al. [44], and Goltsev et al. [42].

Parameters extracted directly from fluorescence measurement were:

**F_O_**—Minimum fluorescence, when all PSII RCs were open;

**F_M_**—Maximum fluorescence, when all PSII RCs were closed;

**F_50__μ__s_, F_100__μ__s_, F_300__μ__s_, F_2ms_, F_30ms_**—Fluorescence intensities at 50, 100, and 300 μs, and 2 and 30 ms, respectively;

**Area**—Total complementary area between fluorescence induction curve and *F = F_m_*;

Parameters calculated from fluorescence measurement data were:

**Fv**—Variable fluorescence; *Fm − F*_0_;

**Fv/Fm**—Maximum quantum yield of PSII; *(Fm – F*_0_*)/Fm*;

**Fv/Fo**—Activity of the water-splitting complex on the donor side of the PSII; *(Fm F*_0_)*/F*_0_;

**V_J_**—Relative variable fluorescence at 2 ms (J-step); *V_J_ = (F_2ms_ – F*_0_*)/(Fm − F*_0_*)*;

**V_I_**—Relative variable fluorescence at 30 ms (I-step); *V_I_ = (F_30ms_ − F*_0_*)/(Fm − F*_0_*)*;

**S_m_**—Normalised total complementary area above the OJIP transient (reflecting multiple-turnover *Q_A_* reduction events) or total electron carriers per RC; *S_m_ = Area/(Fm − F*_0_*)*;

Moreover, the following yield or flux ratios were calculated:

**φ_Po_**—Maximum quantum yield of primary photochemistry at *t* = 0; *φ_Po_ =* 1 *− F*_0_*/Fm = Fv/Fm*;

**φ_Eo_**—Quantum yield for electron transport at *t* = 0; *φ_Eo_ = (Fv/Fm)(*1 *− V_J_)*;

**ψ_Eo_**—Probability (at time 0) that the trapped exciton moves an electron into the electron transport chain beyond; *ψ_Eo_ =* 1 *− V_J_*;

**ρ_Ro_**—Efficiency with which a trapped exciton can move an electron into the electron transport chain from *Q_A‾_* to the PSI and electron acceptors; *ρ_Ro_ = ψ_Eo_δ_Ro_ = (*1 *− V_J_)(*1 *− V_I_)/(*1 *− V_J_)*;

**δ_Ro_**—Efficiency with which an electron can move from the reduced intersystem electron acceptors to the PSI end electron acceptors; *δ_Ro_ = RE_o_/ET_o_ = (*1 *− V_I_)/(*1 − *V_J_)*;

**φ_Ro_**—Quantum yield for the reduction of end acceptors of PSI per photon absorbed; *φ_Ro_ = RE_o_/ABS = φ_Po_ψ_Eo_δ_Ro_*;

Furthermore, the following specific fluxes or activities per RC were calculated:

**ABS/RC**—Absorption flux per RC; *ABS*/*RC* = *Mo*/*V_J_* = 4(*F*_300__μ__s_ − *F*_0_)/(*Fm* − *F*_0_)*/V_J_*;

**TR_o_/RC**—Trapped energy flux per RC at *t* = 0; *TR_o_/RC = Mo/V_J_*;

**ET_o_/RC**—Electron transport flux per RC at *t* = 0; *ET_o_/RC = (Mo/ V_J_)ψ_Eo_*;

**DI_o_/RC**—Dissipated energy flux per RC at *t* = 0; *DI_o_/RC = ABS/RC – TR_o_/RC*;

Additionally, the following phenomenological fluxes or activities per excited cross section (CS). as well as density of RCs were calculated:

**TR_o_/CS_o_**—Trapped energy flux per CS at *t* = 0; *TR_o_/CS_o_ = (ABS/CS_o_)φ_Po_*;

**ET_o_/CS_o_**—Electron transport flux per CS at *t* = 0; *ET_o_/CS_o_ = (ABS/CS_o_)φ_Eo_*;

**DI_o_/CS_o_**—Dissipated energy flux per CS at *t* = 0; *DI_o_/CS_o_ = ABS CS_o_ − TR_o_/CS_o_*;

**RC/CS_o_**—Amount of active PSII RCs per CS at *t* = 0; *RC/CS_o_ = φ_Po_(ABS/CS_o_)(V_J_/Mo).*

In addition, the total performance index was calculated:

**PI_Total_** the integral functional activity of PSII, PSI, and intersystem electron transport chain;

*PI_Total_ = PI_ABS_(δ_Ro_/*1 *− δ_Ro_)*;

#### 4.3.3. Gas Exchange Measurement

Gas exchange measurements were carried out while using a portable gas-exchange systems LCpro-SD (ADC BioScientific Ltd., Hoddesdon, UK) on both leaves and stems of grass pea seedlings. Measurements of net photosynthesis (Pn) were done under CO_2_ saturated conditions (650 μmol·mol^−1^): 300 μmol·s^−1^ of air flow, 50–55% relative humidity within the cuvette, organ temperature of 25 °C, and under 100 μmol [quanta] m^−2^·s^−1^ red light intensity. The stomatal conductance (*G*s) and transpiration rate (*E*) were also measured, and the light compensation point (*L*) was calculated. Photosynthetic light response curves were done on the same plants that were used for net photosynthesis for a stepwise reduction of photosynthetic active radiation (PAR), ranging from 2000 to 0 μmol (quanta) m^−2^·s^−1^ [in 300, 120, 50, 20, 0, 100, 300, 500, 1000, 1500, 2000, 300, and 120 μmol (quanta) m^−2^·s^−1^ steps]. The leaves were adapted to each of the light intensities for 3, 2, 2, 1, 3, 3, 3, 3, 5, 5, 5, 5, and 3 min, respectively, before data point recording.

### 4.4. Evaluation of Photosynthetic Apparatus Rearrangement under NaCl Stress

#### 4.4.1. Protein Content Determination

The Western Blot technique was used in order to determine the presence and content of chosen proteins. Plant tissues (leaves and stem, separately) were extracted in denaturing buffer (100 mM Tris-HCl, pH 8.0, 10% sucrose, 0.2% β-mercaptoethanol and 2% PVPP) according to Laureau et al. [141]. Protein concentration in the extract was determined while using the Bradford reagent and bovine serum albumin (BSA) as a standard [142]. SDS-PAGE electrophoresis was performed at 4 °C, with 24 mA for 15 min, 34 mA for 35 min, and 68 mA for 60 min, on 12% polyacrylamide gels using a vertical gel electrophoresis system (Mini-PROTEAN^®^ Tetra Vertical Electrophoresis Cell, Bio-Rad, Hercules, CA, USA). After electrophoresis, proteins from the polyacrylamide gel were electroblotted onto a nitrocellulose membrane (pore size 0.2 µm) with a semidry electroblotter (Trans-Blot SD Semi-Dry Transfer Cell, Biorad, CA, USA) while using a transfer buffer containing 48 mM Tris (pH 9.2), 39 mM glycine, 20% methanol, and 1.3 mM SDS. Electro-transfer was performed at 10 V (limiting parameter) and 400 mA at room temperature for 30 min. The membranes were blocked at room temperature for 2 h in TBST buffer containing 3% dry milk and then probed with the rabbit primary antibody (Ab) against ascorbate peroxidase (APX, AS08 368, Agrisera, Vinnas, Sweden), catalase (CAT, AS09 501, Agrisera), plastocyanin (PC, AS06 141, Agrisera), and the RuBisCo large subunit (RbcL, AS03 037, Agrisera). After washing, the blot was incubated with horseradish peroxidase-conjugated anti-rabbit secondary antibody (AS09 602, Agrisera) 1:10,000 dilution in TBST buffer containing 1% dry milk for 1.5 h. After washing with TBST buffer, the solution of BCIP and NBT, which was prepared in a buffer containing 100 mM Tris (pH 9.5) and 100 mM NaCl, 5 mM MgCl_2_, was used in order to detect the antigen-antibody complexes. The membranes were next digitalised by the Epson Perfection V750 Pro scanner. The scanned membranes were subjected to densitometry analysis by ImageJ software (version 1.52n, open-source software, NIH, USA). The content of each protein was expressed as arbitral units, being defined as the area under the curve. The values for area were calculated in relation to the maximal area value for each gel expressed as 1.

#### 4.4.2. Chloroplast Isolation

The chloroplasts were isolated from freshly collected leaves and stems, separately. Tissues were homogenised in isolation buffer (170 mM Na_2_HPO_4_, 66.7 mM KH_2_PO_4_, 400 mM saccharose, 600 mM NaCl, 300 mM MgCl_2_ × 6H_2_O, 0.1% BSA, 7 mM ETDA) on ice. After filtration through a 100 μm nylon-mesh filter, the homogenate was centrifuged (7 min, 700 rpm, 4 °C). The collected supernatant was centrifuged for 25 min at 2500 rpm and 4 °C. After being resuspended in isolation buffer, the pellet was overlaid on a saccharose gradient (45% saccharose over 60% saccharose) and then centrifuged (25 min, 4500 rpm, 4 °C). Viable chloroplasts, visible as a ring on the border of the two solutions, were collected, suspended in isolation buffer, and freeze-dried for 48 h.

#### 4.4.3. Blue-Native Electrophoresis

The freeze-dried chloroplasts were resuspended in two volumes of the 3× ACA buffer (2.25 M aminocaproic acid, 150 mM Bis-Tris, 1.5 mM EDTA, pH 7.0). The protein concentration was determined with the Bradford assay [142]. The chloroplast membrane complexes were solubilised with 10% *n*-Dodecyl β-D-maltoside (DDM) solution that was prepared in 1× ACA buffer (0.75 aminocaproic acid, 50 mM Bis-Tris, 0.5 mM EDTA, pH 7.0) (final concentration: 0.75% DDM and 1.3 μg/μL protein). After 15 min of incubation on ice, the preparations were centrifuged at 20,000× *g*. The supernatants were next supplemented with Coomassie blue solution (5% Serva Blue G250, 750 mM aminocaproic acid) at the ratio of 2.2 μL for 12 μL of protein sample and then loaded onto the gel. Blue-native PAGE was performed according to Jänsch et al. [143]. Electrophoresis was carried out at 4 °C in the vertical unit TV100YK from Scie-Plas while using a linear gradient of polyacrylamide gel ranging from 5 to 12%. The protein complex separation was performed at 80 V until the dye front reached the separating gel and continued at 100 V for 8 h. Afterwards, electrophoresis gels were digitalised using the Epson Perfection V750 Pro flatbed scanner.

#### 4.4.4. ATPase Activity Assay

After BN-PAGE, the gels were equilibrated in reaction buffer (35 mM Tris, 270 mM glycine, 14 mM MgSO_2_, 0.2% Pb(NO_3_)_2_, and 8 mM ATP, pH 7.8) without reagents for 10 min This equilibration step was repeated three times, each time changing the buffer for a fresh one. The gels were next transferred to a staining solution [144] and then incubated for 16 h. After staining, the gels were digitalised while using an Epson Perfection V750 Pro scanner. Optical density (OD) was measured using the ImageJ software (v.1.52a, Rasband 1997–2018). These OD values were normalised by the OD values of the LHCII trimer band measured directly after BN-PAGE.

#### 4.4.5. Second Dimension Electrophoresis

Tris-tricine SDS-PAGE was carried out according to Shägger and von Jagow [145] while using the vertical gel unit TV400K from Scie-Plas. After the in-gel activity assay, the gels were incubated in denaturing solution (2% SDS, 1% β-mercaptoethanol, 62.5 mM Tris, pH 6.8) at 56 °C for 10 min Subsequently, single lanes were cut out and then placed horizontally side-by-side on top of the stacking gel; they represented a control plant, and plants that were treated with 50 and 100 mM NaCl, respectively. One µL of the protein molecular weight marker (PageRuler Unstained Protein Ladder, Thermo Scientific, Waltham, MA, USA) was applied onto the pieces of Whatman paper (2 × 5 mM) and placed on top of the stacking gel between BN lanes. Whatman strips and BN lanes were both sealed with agarose solution (0.5% agarose, 750 mM Tris, 2.6 mM SDS, pH 8.4). Electrophoresis was performed at 200 V and 120 mA at 4 °C for 5 h. After electrophoresis, the gels were silver stained according to Jungblut and Seifert [146].

#### 4.4.6. ATR-FTIR Measurements

Freeze-dried chloroplasts from leaves and stems were deposited on ATR crystal. The ATR-FTIR spectra were recorded with a Bruker Alpha FTIR spectrometer with a single-bounce diamond ATR crystal. For each sample, three spectra were acquired with a 4 cm^−1^ spectral resolution in the region of 4000 to 400 cm^−1^ by co-adding 256 scans.

Pre-processing of spectra, analysis, and data presentation were performed while using OPUS (Bruker Optics, Bullerica, MA, USA, Version 7.2.139.1294) and OriginPro 9.1 (ver. 2019b, OriginLab, Northamptin, MA, USA) software. Firstly, the extension of the ATR correction was applied as implemented in OPUS software. After normalisation in the region of 3100–900 cm^−1^, second derivative IR spectra were calculated with nine smoothing points according to the Savitzky–Golay protocol [147]. Second derivative/absorption spectra were used for the calculation of the integral intensity of various bands using the OPUS program. For this purpose, a linear baseline was drawn through the peak edges, and the spectrum that was below this line was integrated over the wavenumber range of the band (Supplementary Materials: Table S2). For the comparison of spectral differences between studied groups, the spectra from each measurement were averaged within the sample.

### 4.5. Statistical Analyses

All of the spectrophotometric estimations were made in five replications. Chl *a* fluorescence measurements were made in ten replications. Gas exchange measurements and electrophoresis were made in three replications. Statistical analyses were done while using STATISTICA 12.0 (StatSoft Inc., Tulsa, OK, USA). The results, within organ and parameter, were subjected to one-way analysis of variance (ANOVA), and the significance of differences between the arithmetical means was determined by Duncan *post hoc* test at *p* ≤ 0.05.

## 5. Conclusions

Severe salt stress (100 mM NaCl) caused a significant sodium translocation to seedling shoots, which leads to disturbances in primary metabolism. Consequently, interferences in the function of the photosynthetic apparatus were observed, which led to an intense increase in the demand for ATP and NADPH. Moreover, the increasing sodium content in the aboveground part of the plants revealed differences in the response of the photosynthetic apparatus in grass pea leaves and stems. Moderate salinity stress induced the protection of leaves photosynthetic apparatus by the activation of APX and CAT, as well as the intensive synthesis of soluble sugars. Conversely, severe salt stress changed the function of the leaves from the source of assimilates to the place of harmful ion (Na^+^) accumulation. In turn, in the stems, severe salt stress triggered the acclimatisation mechanism, which consists of the structural and functional reconstruction of the photosynthetic apparatus. The reconstruction involved a change in the composition of thylakoid membranes (increase in the degree of fatty acid unsaturation, decrease in alpha-helical protein structures) and the size and method of protein association of both antenna and photosystem complexes. As a result, a functional basis was created for the activation of alternative electron transport pathways, including cyclic, which resulted in an increase of ATP synthesis, secured the uncontrolled production of ROS, and enabled the effective carboxylation of CO_2_. Consequently, stems took over the function of the main source of assimilates from the current production. The observed structural and functional changes of grass pea photosynthetic apparatus enable an effective mechanism of tolerance to salinity stress.

## Data Availability

Not applicable.

## Supplementary Materials

Supplementary materials can be found at https://www.mdpi.com/1422-0067/22/2/685/s1.

## Abbreviations

ALAD	alpha amino levulinic dehydrogenase
APX	ascorbate peroxidase
ATR-FTIR	attenuated total reflectance—Fourier-transform infrared
BCIP	5-bromo-4-chloro-3-indolyl phosphate
BN-PAGE	blue-native polyacrilamide gel electrophoresis
BSA	bovine serum albumin
CAT	catalase
CET	cyclic electron transport
CS	cross section
DW	dry weight
EDTA	ethylenediaminetetraacetic acid
FRAP	ferric reducing antioxidant power
LET	linear electron transport
Lhca1-3	Photosystem I type light-harvesting chlorophyll a/b binding proteins
Lhcb1-3	Photosystem II light-harvesting chlorophyll a/b binding proteins
LHCI	Light-harvesting complex of PSI
LHCII	Light-harvesting complex of PSII
MDA	malondialdehyde
NADPH	reduced nicotinamide adenine dinucleotide phosphate
NBT	nitro blue tetrazolium
NDH	NAD(P)H dehydrogenase complex
ODAP	β-N-oxalyl-L-α,β-diamino propionic acid
OEC	oxygen evolving complex
PAR	photosynthetic active radiation
PC	plastocyanin
PME	pectin methyl esterase
PQ	plastoquinone
PetA, C, D	Protein subunits of cytochrome *b6f* complex
PsaA, B	core proteins of photosystem I
PsaD, E, F, L	Protein subunits of photosystem I
PsbA, D	Photosystem II reaction center protein D1 and D2
PsbB	CP47 protein of photosystem II
PsbC	CP43 protein of photosystem II
PsbS	Lhc-like PSII protein
PsbO	oxygen-evolving enhancer protein 1
PSI	Photosystem I
PSII	Photosystem II
PTOX	terminal plastoquinone oxidase
RC	reaction center
RbcL	rubisco large subunit
ROS	reactive oxygen species
Rubisco	ribulose-1,5-bisphosphate carboxylase
SDS-PAGE	sodium dodecyl sulfate–polyacrylamide gel electrophoresis
TBA	thiobarbituric acid
TCA	trichloroacetic acid
