# Supplementary material for: Stem Photosynthesis—A Key Element of Grass Pea (Lathyrus sativus L.) Acclimatisation to Salinity
Source: Int J Mol Sci. 2021 Jan 12;22(2):685. doi: 10.3390/ijms22020685 (PMC7828162; doi:10.3390/ijms22020685)
Supplement: Supplementary file 1 [file ijms-22-00685-s001.pdf]

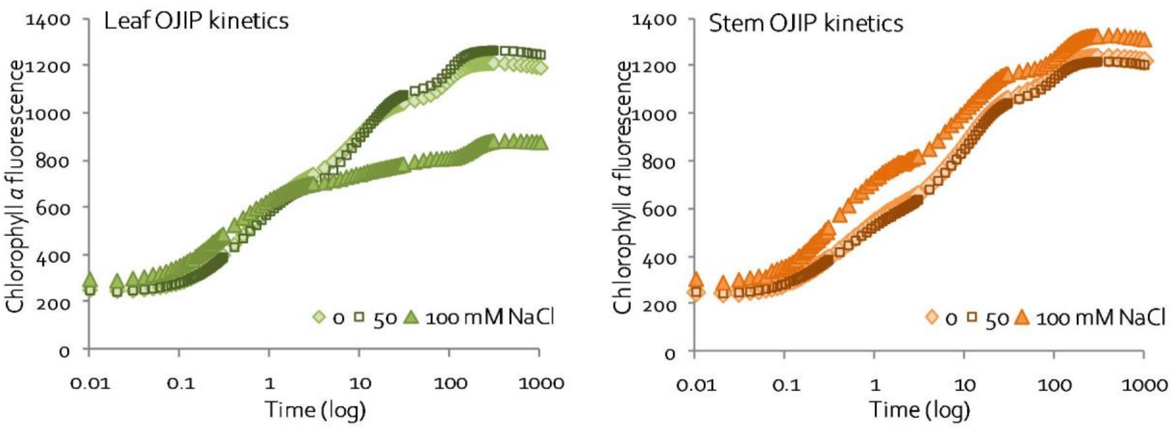

Figure S1. Reaction of grass pea leaf and stem photosynthetic apparatus to salinity. The fast chlorophyll *a* fluorescence transient (OJIP kinetics) of grass pea leaves and stems after 25 days of cultivation under 0, 50 and 100 mM NaCl

Table S1. Raw value of Fv/Fm (the maximum quantum yield of PSII) of grass pea leaf and stem photosynthetic apparatus after 25 days of cultivation under 0, 50 and 100 mM NaCl

NaCl [Mm]	Fv/Fm value	
	Leaf	Stem
0	0.81^{a} ±0.01	0.81^{a} ±0.01
50	0.82^{a} ±0.01	0.81^{a} ±0.01
100	0.78^{b} ±0.05	0.81^{a} ±0.01

Table S2. FTIR bands assignment to biomolecules (ν – stretching and δ - bending modes) [49-53].

	IR band position [cm^{-1}]	Assignment
LIPIDS	3012	=C-H, ν(=CH)
	2960	$-CH_3$, ν(-C-H)
	2936-2924	$-CH_2$, ν_{as}(-C-H)
	2855-2851	$-CH_2$, ν_{s}(-C-H)
	1740	Triacylglycerols, ν(C=O)
	1716	Free fatty acids, ν(C=O)
PROTEINS	1680	Amide I, β-turn, ν(C=O)
	1654	Amide I, α-helix, ν(C=O)
	1633	Amide I, β-sheet, ν(C=O)
	1548/1541	Amide II, δ(N-H)/ν(C-N)
	1516	Tyrosine
SACCHARIDES	1076	Saccharides, ν(C-O)/ν(C-C)
	1048	Saccharose, fructose, ν(C-O)
	1021	Saccharides, ν(C-O)
	990	Starch, saccharose, glucose, ν(C-O)/ν(C-C)
	944	Saccharose, $\delta(CH_2)$
	921	Saccharides, δ(CCO)

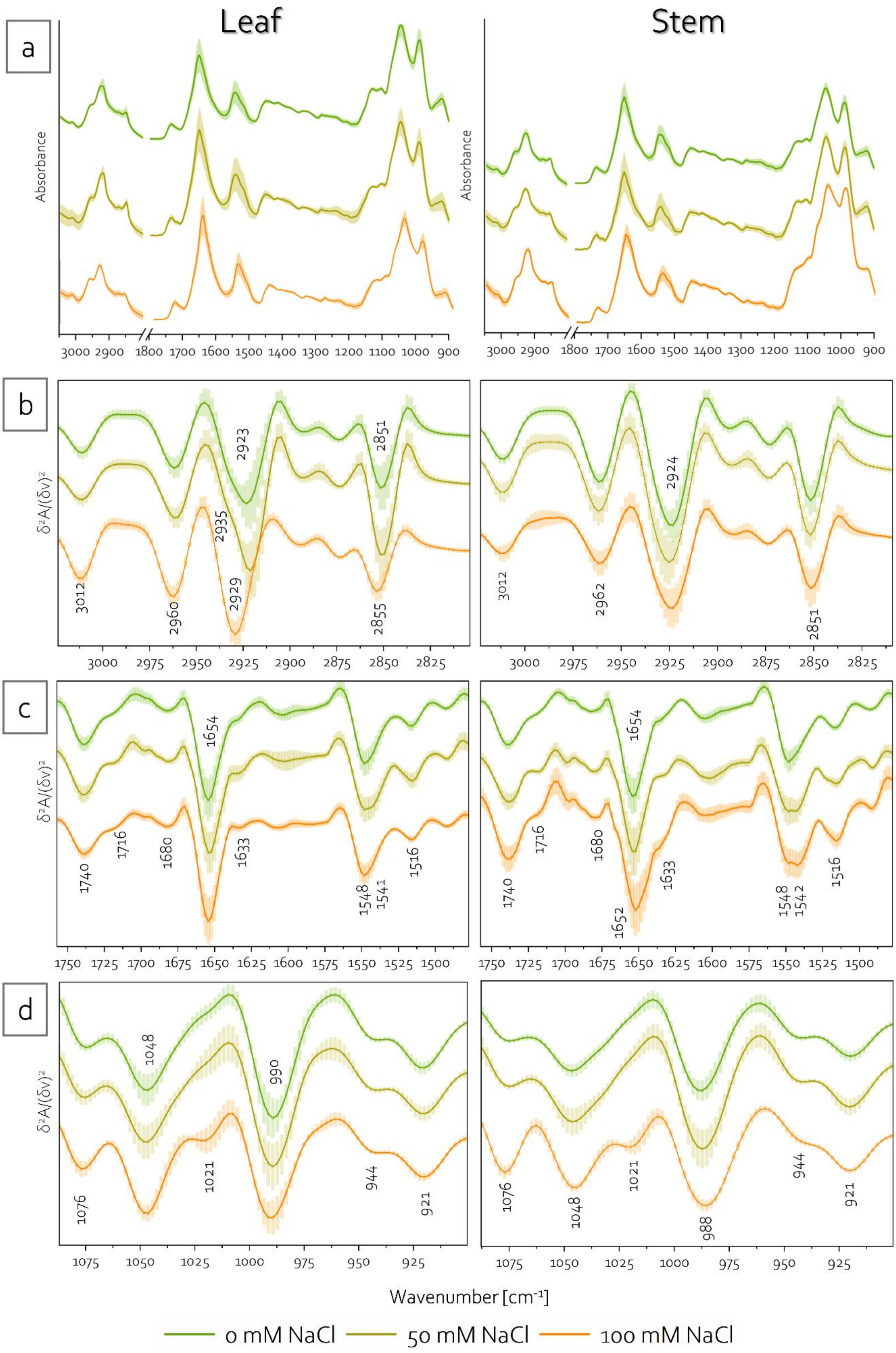

Figure S2. Averaged raw ATR FTIR spectra (a) and their second derivatives with peak labelling in the regions specific for (b) fatty acids (3050 – 2800 cm^{-1}), (c) triacylglycerols, fatty acids and proteins (1750 – 1480 cm^{-1}) and (d) sugars (1100 – 900 cm^{-1}). Shading of spectra denotes standard deviation (n = 3).
